# Long noncoding RNA GDIL acts as a scaffold for CHAC1 and XRN2 to promote platinum resistance of colorectal cancer through inhibition of glutathione degradation

**DOI:** 10.1038/s41419-025-07374-w

**Published:** 2025-02-01

**Authors:** Xuan Deng, Lu Chang, Lingyu Tang, Haoqin Jiang, Xiao Xu, Xinju Zhang, Jian Chen, Liu Dong, Qianqian Xu, Ruoshui Cao, Jianbin Xiang, Ming Guan

**Affiliations:** 1 Department of Laboratory Medicine, Huashan Hospital Fudan University, Shanghai, 200040 China; 2 Department of Gastroenterology, Huashan Hospital, Fudan University, Shanghai, 200040 China; 3 Department of General Surgery, Huashan Hospital, Fudan University, Shanghai, 200040 China

**Keywords:** Cancer therapeutic resistance, Colorectal cancer

## Abstract

Acquired resistance poses a significant obstacle to the effectiveness of platinum-based treatment for cancers. As the most abundant antioxidant, glutathione (GSH) enables cancer cell survival and chemoresistance, by scavenging excessive reactive oxygen species (ROS) induced by platinum. Therapeutic strategy targeting GSH synthesis has been developed, however, failed to produce desirable effects in preventing cancer progression. Thus, uncovering mechanisms of rewired GSH metabolism may aid in the development of additional therapeutic strategies to overcome or delay resistance. Here, we identify upregulation of long noncoding RNA (lncRNA) GDIL (GSH Degradation Inhibiting LncRNA) in platinum resistant colorectal cancer (CRC) and ovarian cancer cells compared with parental ones. High expression of GDIL in resistant CRC is associated with poor survival and hyposensitivity to chemotherapy. We demonstrate that GDIL boosted GSH levels and enhances clearance of ROS induced by platinum. Metabolomic and metabolic flux analysis further reveals that GDIL promotes GSH accumulation by inhibiting GSH degradation. This is attributed by downregulation of CHAC1, an enzyme that specifically degrades intracellular GSH. Mechanistically, GDIL binds and re-localizes the nuclear protein XRN2 to the cytoplasm, where GDIL further serve as a scaffold for XRN2 to identify and degrade CHAC1 mRNA. Suppression of GDIL with selective antisense oligonucleotide, restored drug sensitivity in platinum resistant cell lines and delayed drug resistance in cell line- and patient-derived xenografts. Thus, lncRNA GDIL is a novel target to promote GSH degradation and augment platinum therapy.

## Introduction

One important mechanism that cancer cells possess to resist chemotherapy is to migitate the lethal damage caused by drug-induced reactive oxygen species (ROS), via highly active antioxidant systems [1, 2]. Glutathione (GSH), the most abundant intracellular antioxidant, is increased in tumor tissues less sensitive to treatments [3]. Blocking antioxidant production, particularly GSH synthesis, has been considered as a promising approach for cancer treatment [4]. L-buthionine-sulfoximine (BSO) is an inhibitor of the rate-limiting enzyme (GCLC) for GSH de novo synthesis. The administration of BSO has been demonstrated to prevent tumor initiation in several mice models of spontaneous carcinogenesis [5]. Nevertheless, the impact on well-established tumors has been somewhat limited, most only result in partial exhaustion of GSH and therefore have not found clinical success. [5]. Harris et al. also showed that majority of cancer cell lines exhibited no decrease in cell count following GSH depletion by BSO [6]. These facts indicate regulation diversity of GSH metabolism, suggesting that other pathways may resist GSH depletion by BSO. Moreover, the non-selective nature of GSH-depleting agents may cause irreversibly damaged to non-malignant tissues. Thus, identification of novel pathways regulating GSH metabolism during cancer progression may help identifying novel targets with enhanced cancer specificity.

Increased GSH levels can also be a byproduct of cancer cell metabolic reprogramming [7]. GSH degradation is a key determinant of GSH homeostasis, in addition to its biosynthesis [8]. However, the contribution of GSH degradation to chemoresistant phenotypes, remains unclear. In this study, we investigated transcriptomic and metabolic networks in paired parental and platinum-resistant cancer cells. We identified a novel lncRNA GDIL (GSH Degradation Inhibiting LncRNA) as a non-coding RNA with upregulated expression during platinum resistance in ovarian cancer and CRC, resulting in scavenging of ROS and rewiring of GSH metabolism. Here, we systemically demonstrate that GDIL drives acquired platinum resistance by inhibiting GSH degradation instead of increasing GSH synthesis, through GDIL/XRN2/CHAC1 pathways. We also report that high levels of GDIL is associated with poor survival in CRC patients. Furthermore, the combination of GDIL inhibition and platinum leads to delayed resistance, suggesting the functionality and specificity of GDIL as a therapeutic target.

## Materials and methods

### Cells

Human embryonic kidney 293 T (HEK293T), ovarian cancer cell lines (SKOV3 and OVCAR3) and colorectal cancer cell lines (SW480 and HCT116) were obtained from ATCC (Manassas, VA, USA) and authenticated by cell line STR analysis (Genetic Testing Biotechnology, Jiangsu, China). All cells were cultured in DMEM (HyClone, Cytiva, Marlborough, MA, USA) + 10% FBS (Gibco, Grand Island, NY, USA) and incubated at 37 °C in a humidified atmosphere with 5% CO_2_. Cells were passaged at least 3 times and tested negative for mycoplasma contamination prior to being used in experiments.

### Patients and clinical specimens

Nighty colorectal cancer tissue specimens were collected from Huashan Hospital, Fudan University. The inclusion criteria were (a) patients aged 30–90 years; (b) patients with primary resectable CRC; (c) patients with stage II or III CRC confirmed by postoperative pathology; (d) patients received oxaliplatin-based chemotherapy after CRC resection. The exclusion criteria were as follows: (a) patients with other tumor diseases; (b) patients with a previous cancer treatment history; (c) patients with contraindications for colon or rectum surgery. Response Evaluation Criteria in Solid Tumors (RECIST) was applied as standard of assessment of patient treatment response. Tissue samples were frozen at - 80°C until further use. This study was approved by the Ethics Committees in Huashan Hospital (approval number: 2020-578, date: 2020-03-31). All participation understand what the research is and signed written informed consents. Study protocol was approved by the Ethics Committees in Huashan Hospital and carried out in compliance with the 1975 Helsinki Declaration.

### Transcriptome analysis

Transcriptome analysis of indicated cells was performed as previous described [9]. Total RNA was extracted and sequenced on an Illumina HiSeq 3000 (Ribobio, Guangzhou, China). Quality control of raw data was performed and filtered reads were mapped to huaman genome hg19. Read numbers and RPKM (reads per kilobase per million mapped reads) were calculated. Differentially expressed gene (DEG) (|fold change | >2, *p* < 0.05) were analyzed using edgeR.

### RNAscope assay

Probe specifically targeting GDIL were designed and produced in Advanced Cell Diagnostics (Newark, CA, USA). GDIL signals were detected using the RNAscope 2.5 High Definition (HD) detection kit (Brown). Briefly, Cells were plated in chamber slide with 30% confluence. Cells were fixed in 10% neutral buffered formalin (NBF), then dehydrated and rehydrated. RNAscope hydrogen peroxide and RNAscope Protease III were added on slides, incubate at room temperature. Cells were hybridized with RNAscope Probe in the C1 channel. RNAscope probe were detected using the RNAscope Multiplex Fluorescent Detection Reagents Kit v2 (Bio-Techne R&D Systems, Minneapolis, MN, USA). RNAscope quantification is based on the number of fluorescence probe spots.

### Untargeted metabolomics analysis

For untargeted metabolomics, 1 × 10^7^ cells were washed twice with cold PBS, collected and quenched immediately in liquid nitrogen.

The changes in metabolites were examined by Gas and Liquid chromatography coupled to time-of-flight mass spectrometry (GC/TOF-MS and LC/TOF-MS) based metabolomics carried out in the laboratory of Shanghai Luming Biotechnology Co., Ltd. (Shanghai, China). GC/TOF-MS was performed by Agilent 7890B gas chromatography system and 5977 A MSD system (Agilent Technologies, CA, USA). A 30 m × 0.25 mm × 0.25 μm DB-5MS fused-silica capillary column (Agilent Technologies) was used to separate the derivatives. AnalysisBaseFileConverter, MD-DIAL software were used for raw data exaction and analysis.

Waters-VION IMS QTOF-based metabolomics study was performed by ACQUITY UPLC I-Class system and VION IMS QTOF mass spectrometer with a 1.7 µm ACQUITY UPLC BEH C18 Columns (Waters Corporation, Milford, USA). Metabolites were identified by progenesis QI software (Waters Corporation).

The differential metabolites were selected based on the combination of a statistically significant threshold of variable influence on projection (VIP) values obtained from the OPLS-DA model and *p*-values from a two-tailed Student’s *t*-test on the normalized peak areas. Differential metabolites were selected according to the VIP value (VIP ≥ 1.0), *p*-value (*p*-value < 0.05).

### Targeted metabolomics analysis

Cells were washed by cold 0.9% saline solution three times, each sample was extracted by 500 μL methanol: acetonitrile: water (2:2:1) (v/v/v). Each sample was thoroughly lyophilized and then stored at -80°C.

Targeted metabolomics were analyzed using an Vanquish UPLC system & Q Exactive plus Mass spectrometer (Thermo Fisher Scientific) in Complete Omics Inc (Hangzhou, Zhejiang, China). Data processing were performed using TraceFinder 5.0 (Thermo Fisher Scientific).

### Isotopic tracing analysis

Cells (1 × 10^7^ cells in 10 cm dish) were incubated in cystine-free DMEM medium (Hyclone) containing 0.2 mM 3,3′-^13^C_2_-cystine (IsoReag, IR-30573) for indicated duration. Cells were washed with cold 0.9% saline solution three times, each sample was extracted by 500 μL methanol: acetonitrile: water (2:2:1) (v/v/v). Samples were injected and analyzed by an Vanquish UPLC system & Q Exactive plus Mass spectrometer in Complete Omics Inc.

### Glutathione quantification

Glutathione quantification including GSH and GSSG in cells were calculated using GSH/GSSG-Glo^TM^ Assay (Promega). Cells (1 × 10^4^ cells per well) were seeded into 96-well plates and lysed with total or GSSG reagent. Add luciferin generation reagent. After 30 min incubation, add luciferin detection reagent. Read luminescence using microplate reader (BioTek, Winkowski, VT, USA).

### Colorectal cancer xenograft experiments

Four to 6 week-old female BALB/c nude mice were obtained from Fudan University. All mice were bred under specific pathogen-free (SPF) conditions in flow cabinets. The mice were housed with food and water provided ad libitum.

For CDX models: 2 × 10^6^ HCT116 or HCT116OR cells were inoculated subcutaneously into flank of each mouse to establish the CRC xenograft model. When tumor sizes reach around 200 mm^3^, mice received oxaliplatin (5 mg/kg), cisplatin (5 mg/kg) or vehicle treatment (intraperitoneal injections, i.p.) twice a week for 3 weeks. Oxaliplatin was dissolved and diluted by sterile water. For NAC treatment group, 5 g/liter NAC was supplemented in drinking water (pH 6.6 to 7.2). Tumor size were measured every 3 days. Tumor volume was calculated as long × short^2^ (in millimeter). Mice were sacrificed 3 weeks after drug treatment, and subcutaneous tumors were collected and weighed.

PDX studies were conducted at Shanghai Model Organisms Center Inc. (Shanghai, China) under the instruction and supervision of M.G.’s laboratory. Eight to 10 week-old NOD/SCID mice bearing PDX were randomized into different groups. When tumor size reached 200 mm^3^, PDXs received subcutaneous peritumor injection of vehicle, oxaliplatin (5 mg/kg), control ASO (50 mg/kg) and GDIL-ASO (50 mg/kg) every 3 days. Tumor size were measured every 3 days.

All mouse experiments in this study were complied with the ARRIVE guidelines and were conducted in accordance with the National Research Council’s Guide for the Care and Use of Laboratory Animals. The study procedures were approved by the Committee on the Ethics of Animal Experiments of Fudan University. The maximum allowable tumor size for a single implanted tumor that is visible without imaging is ~2 cm in any dimension in adult mice. We confirmed that the maximal tumor size/burden was not exceeded that criteria.

### Biotin pull-down assays and mass spectrometry

Wild type (sense), antisense, and CHAC1 binding site mutated GDIL RNA were in vitro transcribed and biotinylated with RNAmax-T7 Biotin RNA Labeling Kit (Ribobio). RNAs and proteins in cell lysates were incubated with biotinylated GDIL and purified by streptavidin agarose beads (Invitrogen). GDIL binding RNAs were detected via qRT-PCR. GDIL binding proteins were identified by immunoblotting.

### MS2 pull-down assay

Cells (5 × 0^7^) were co-transfected with pMS2-GST, pGDIL-MS2 vector or pEmpty-MS2 plasmids using Lipofectamine 3000 for 48 h. Cells were lysed, proteins are extracted and incubated with GSH agarose (Millipore Sigma) for 5 h at 4°C. Unspecific bound proteins were detached by 3 times of washing with cell-lysis buffer. Proteins interacted with GDIL were detected by immunoblotting.

### RNA immunoprecipitation

Cell lysates were collected and incubated with anti-XRN2 antibody (Cell Signaling Technology Beverly, MA, USA) and Protein G Magnetic Beads (Thermo Fisher Scientific) for 4 h at 4°C. Beads were washed 3 times with lysis buffer and mixed with RLT buffer. Then XRN2-interacting RNAs were purified by RNeasy mini kit (Qiagen) and detected by qRT-PCR.

### Fluorescence in situ hybridization (FISH) and Immunofluorescence

Cells were seeded in cell culture chamber slides and fixed with 4% paraformaldehyde. Then 0.5% Triton X-100 was added at 37 °C for 30 min for permeabilization. GDIL FISH signal were detected using a Fluorescent In Situ Hybri-dization (FISH) Kit (RiboBio). Cells were incubated with GDIL probe (designed and produced in Ribobio) at 37 °C overnight. After washing with SSC, cells were incubated with XRN2-antibody at room temperature for 1 h. Finally, Alexa Fluor 488-conjugated secondary antibody (Abcam, ab150077, Cambridge, MA, USA) and DAPI were added. Images was acquired with a Leica stellaris 5 confocal laser-scanning microscope.

### Statistical analysis

All experiments were performed in triplicate (or more). Data was presented as mean ± SEM. Statistic analysis were performed using *t*-tests and Chi-square tests. The data were analyzed by SPSS (IBM, NY, USA) or GraphPad Prism (GraphPad Software, CA, USA). Nonlinear regression analysis was plotted between drug concentration and cell inhibition percentage. Differentially expressed genes from transcriptome analysis were analyzed using edgeR. IC_50_ was determined by GraphPad Prism. Results are statistically significant when *p*-values are <0.05 (*p < 0.05, **p < 0.01, and ***p < 0.001).

## Results

### GDIL upregulation in oxaliplatin resistant CRC cells

**Fig. 1:**
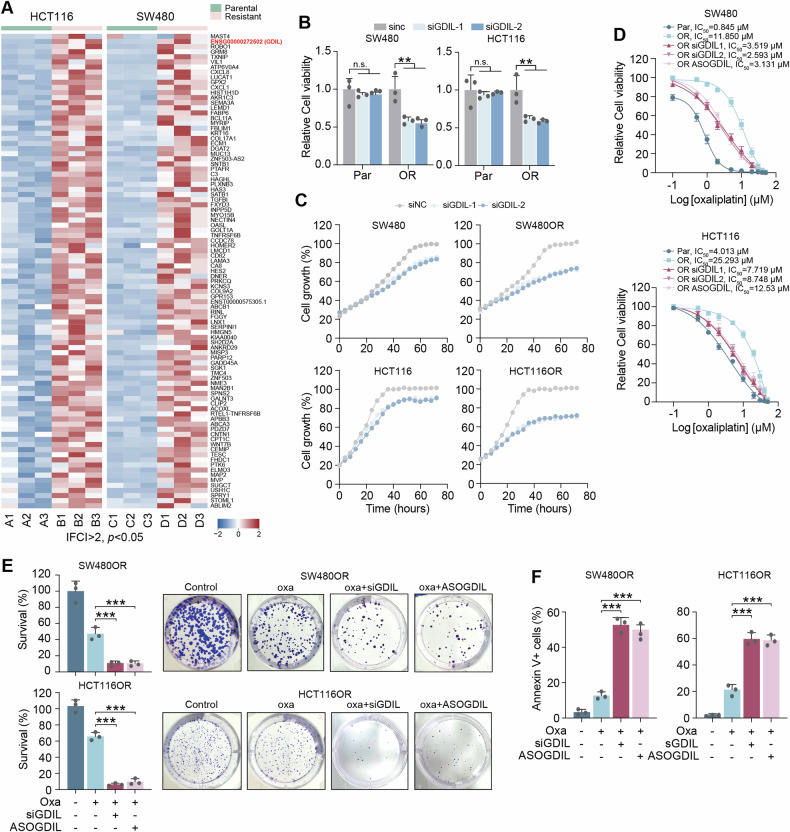
Knockdown of GDIL re-sensitize resistant cells to oxaliplatin therapy. **A** Heatmap showing transcripts upregulated in both oxaliplatin resistant HCT116 and SW480 cells compared with their parental cells analyzed by RNA sequencing (|fold change | >2, p < 0.05). Relative expression is scaled as -2 to 2. **B**, **C** Relative cell viability (**B**) and growth (**C**) in parental (Par) and oxaliplatin resistant (OR) SW480 and HCT116 cells with GDIL knockdown by siRNAs. **D** Relative cell viability of SW480 and HCT116 cells under oxaliplatin treatment with indicated concentrations. GDIL expression was silenced by siRNAs or ASO. **E** Colony formation were analyzed in SW480OR (oxaliplatin: 12 μM) and HCT116OR (oxaliplatin: 25 μM) cells after GDIL knockdown. **F** Apoptosis were analyzed in SW480OR (oxaliplatin: 3 μM) and HCT116OR (oxaliplatin: 8 μM) cells after GDIL knockdown.

HCT116OR, SW480OR, C4OR and R21OR are colorectal cancer (CRC) cell lines that have developed resistance to oxaliplatin [10]. These cell lines were generated from the HCT116, SW480, and patient-derived C4 and R21 cells, respectively. Transcriptome (RNA-seq) analysis showed that in total 86 mRNAs and 7 long non-coding RNAs (lncRNAs) were upregulated in HCT116OR and SW480OR resistant cells (|fold change | >2, *p* < 0.05; Fig. 1A). Reverse transcription quantitative polymerase chain reaction (RT-qPCR) validation increased expression of 7 out of the top 10 genes in C4OR and R21OR cells compared with parental ones (Supplementary Fig. 1A). Each of the 7 genes was knocked down individually using specific small interfering RNAs (siRNAs), and we examined the effects on cell survival. Among the 7 candidate genes, knockdown of ENSG00000272502 (renamed as GSH Degradation Inhibiting LncRNA, GDIL) most drastically decreased cell viability under oxaliplatin treatment (Supplementary Fig. 1B). Besides, Silencing LUCAT1 was found to resensitize cells to oxaliplatin (Supplementary Fig. 1B), confirming findings of an earlier report and also the robustness of this study to identify key lncRNA regulators [11].

### Knockdown of GDIL re-sensitize resistant cells to oxaliplatin therapy

Before we further validate the role of GDIL in chemoresistance, characteristics of GDIL need to be determined. Full-length sequence of GDIL was obtained by 5’ and 3’ rapid amplification of complementary DNA ends (RACE) assay (Supplementary Fig. 1C). GDIL is composed of one exon with a full-length of 1141 nt, without typical protein-coding open reading frames (ORFs) >300nt (Supplementary Fig. 1D and Supplementary Table 1). The non-coding nature of GDIL was also confirmed by coding-potential analysis (Supplementary Fig. 1E, Supplementary Tables 2 and 3). We separated the nuclear and cytoplasmic fractions of cancer cells and measured GDIL’s subcellular localization by qRT-PCR. Considerable enrichment of GDIL was found in the cytoplasm versus the nucleus (Supplementary Fig. 1F). Moreover, fluorescence in situ hybridization (FISH) by RNAscope showed that GDIL was significantly increased in the cytoplasm of oxalipltin resistant cells (Supplementary Fig. 1G). Consistently, increase of GDIL abandunce was observed in oxalipltin resistant CRC cells (246–332 copies/cell for resistant and 57–89 for parental) (Supplementary Fig. 1H).

In order to investigate the reliance of the resistant cell lines on GDIL, we assessed cell survival and proliferation after the genetic or pharmacological inhibition of GDIL using siRNAs or antisense oligonucleotide (ASO) (Supplementary Fig. 2A). SW480OR, and HCT116OR cells exhibited increased sensitivity to GDIL knockdown, despite their resistance to oxaliplatin as compared to their parental cells (Fig. 1B–D, Supplementary Fig. 2B–D). Furthermore, inhibition of GDIL restored the susceptibility of these resistant cells to oxaliplatin. (Fig. 1D). Consistently, this was recapitulated by cell growth, colony formation and apoptosis analysis (Fig. 1E and F, Supplementary Fig. 2E).

### GDIL induces resistance to multiple generations of platinum

**Fig. 2:**
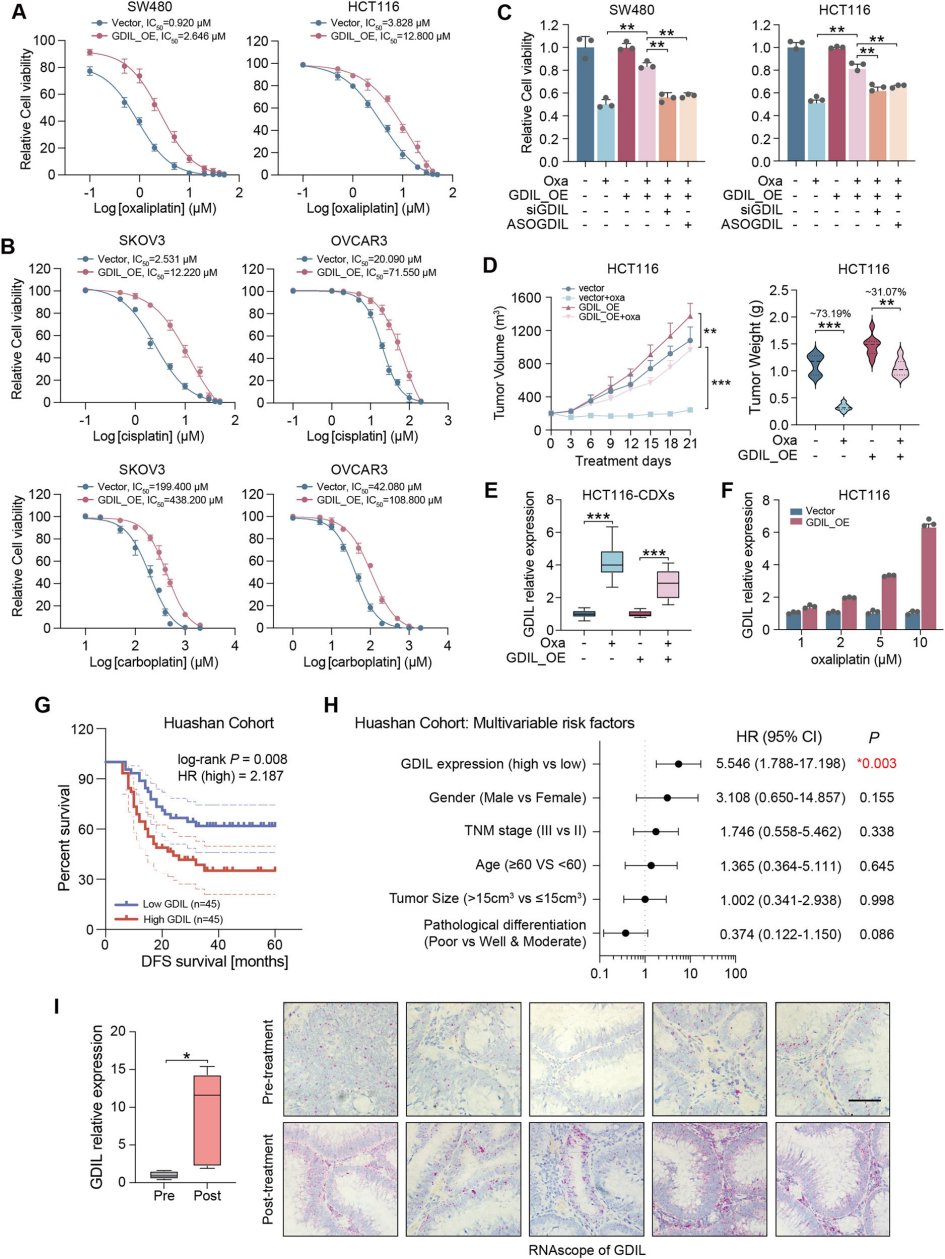
GDIL induces resistance to multiple generations of platinum. **A** Relative cell viability of SW480 and HCT116 control (vector) and GDIL overexpression (GDIL_OE) cells under oxaliplatin treatment with indicated concentrations. **B** Relative cell viability of SKOV3 and OVCAR3 control (vector) and GDIL overexpression (GDIL_OE) cells under cisplatin and carboplatin treatment with indicated concentrations. **C** Relative cell viability of SW480 and HCT116 cells under oxaliplatin treatment (10 μM). **D** Control and overexpression of GDIL in HCT116-CDX treated with oxaliplatin (5 mg/kg) shown in tumor growth (left) and weight change (right). **E** Relative GDIL expression in CDXs tissues from **D**. **F** Relative GDIL expression in HCT116 cells treated with indicated concentrations of oxaliplatin. **G** Disease-Free Survival (DFS) was compared between patients with low and high expression of GDIL in Huashan Cohort, Log-rank test. **H** Multivariate analysis was performed in Huashan Cohort. The bars correspond to 95% confidence intervals. **I** Expression comparisons of GDIL examined by qRT-PCR (left) and RNAscope (right) between paired pre- and post-oxaliplatin treatment CRC samples for those who developed recurrence. Scale bar, 100 μm.

Based on previous results, we demonstrated the dependence of GDIL in platinum resistance. This was confirmed by showing that overexpression of GDIL in SW480 and HCT116 cells significantly conferred resistance to oxaliplatin (Fig. 2A and Supplementary Fig. 3A). Oxaliplatin is the third-generation of platinum. We then investigate whether GDIL is also involved in developing resistance to the first- and second- platinum, cisplatin and carboplatin. Increased resistance to cisplatin and carboplatin was observed, in ovarian cancer cell lines SKOV3 and OVCAR3 with ectopic GDIL expression (Fig. 2B and Supplementary Fig. 3A). The platinum resistance caused by GDIL overexpression was eliminated by inhibition of GDIL (Fig. 2C and Supplementary Fig. 3B). GDIL promotes platinum resistance was also confirmed by in vivo models. Growth of control HCT116 cell-derived xenografts (CDXs) were inhibited by oxaliplatin treatment, while GDIL overexpression CDXs induced drug resistance phenotypes (Fig. 2D). Tumors exhibited an increase in the expression of GDIL as a result of ongoing oxaliplatin therapy (Fig. 2E), consistent with the in vitro models where GDIL gradually upregulated when cancer cells developing acquired resistance to oxaliplatin (Fig. 2F). These findings were also observe in SKOV3 CDXs under cisplatin treatment (Supplementary Fig. 3C–E).

Fresh tumor samples were collected from 90 CRC patients and divided into two groups with high (*n* = 45) and low (*n* = 45) GDIL expression (Supplementary Table 4). We found that GDIL levels positively correlated with the risk of death in CRC (Fig. 2G). High levels of GDIL were strongly associated with shorter DFS (Fig. 2H). More importantly, comparisons between paired pre- and post-oxaliplatin treatment CRC samples for those who developed recurrence (*n* = 5), GDIL expression was activated during chemotherapy (Fig. 2I). These findings suggests that GDIL promotes platinum resistance and is a potential biomarker for CRC recurrence.

### GDIL inhibits intracellular GSH degradation

**Fig. 3:**
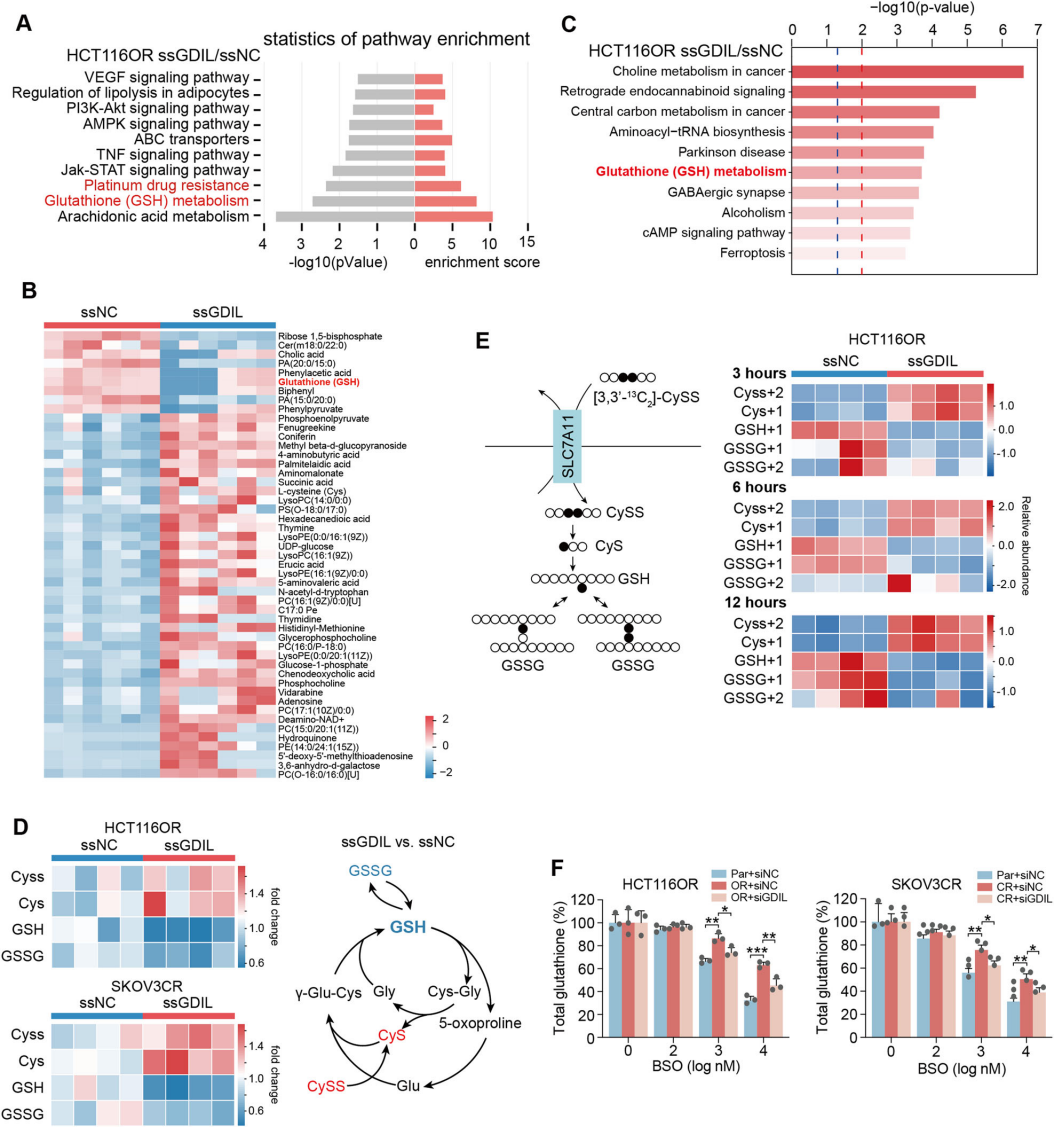
GDIL inhibits intracellular GSH degradation. **A** KEGG enrichment analysis of genes whose expression was significantly affected by GDIL knockdown in HCT116OR cells (ssNC: negative control of smart silencer, ssGDIL: smart silencer targeting lncRNA GDIL). **B** Differential metabolites in control (ssNC) and GDIL knockdown (ssGDIL) HCT116OR cells. Relative abundance is scaled as -2 to 2 (*n* = 6). **C** Enrichment analysis of the top 10 pathways associated with differential metabolites. **D** Left: Heatmap shows relative abundance of metabolites in GSH metabolism pathway in control (ssNC) and GDIL knockdown (ssGDIL) HCT116OR and SKOV3OR cells, *n* = 4 per group. CySS, cystine, the oxidized cysteine; CyS, cysteine; GSH, reduced glutathione; GSSG, oxidized glutathione. Right: schematic (right) shows abundance change of metabolites in GSH metabolism pathway. Red: metabolites increase, blue: decrease. **E** Left: schematic illustrating 3,3′-^13^C_2_-cystine flux to GSH synthesis. Dark circles: ^13^C-labeled carbons; empty circles: ^12^C-carbons. Right: Cystine flux to GSH synthesis in control (ssNC) and GDIL knockdown (ssGDIL) HCT116OR cells. Isotype tracing was performed using 3,3′-^13^C_2_-cystine, *n* = 4 per group. **F** Total glutathione content in control (ssNC) and GDIL knockdown (ssGDIL) HCT116OR and SKOV3CR cells exposed to 0–10^4 ^nM BSO for 72 h.

Aberrant regulation of gene expression and metabolites abundance is reprted to be involved in cancer progression and chemoresistance [12, 13]. To clarify the underlying mechanism of GDIL’s pro-resistance effect, RNA sequencing and untargeted metabolomic analysis was performed in HCT116OR cells with or without GDIL knockdown. Consistent with our findings, KEGG analysis showed that genes regulated by GDIL knockdown were enriched in platinum drug resistance (Fig. 3A, Supplementary Fig. 4A). Integrated two omics data revealed that GSH metabolism pathway was among the top-ranked differential pathways regulated by GDIL (Fig. 3A–C). Targeted metabolomics confirmed that GSH was downregulated after GDIL knockdown (Fig. 3D). The decreased levels of GSH when silencing GDIL was also validated by in vivo models (Supplementary Fig. 4B).

We next traced the metabolic flux of exogenous 3,3′-^13^C_2_-cystine and found decreased intracellular ^13^C-labeled GSH in GDIL knockown cells (Fig. 3E). To be noticed, cystine uptake or GSH precursor (cystein), were increased (Fig. 3E). By contrast, GDIL overexpression increased ^13^C-labeled cystine-derived GSH abundance but not that of precursors (Supplementary Fig. 4C, D). The intracellular GSH pool is maintained not only by GSH synthesis but also by GSH degradation [14]. We thus hypothesized that GDIL possibly inhibited GSH degradation. Indeed, when GSH synthesis was blocked by BSO, silencing GDIL could further accelerated intracellular GSH depletion (Fig. 3F). While GDIL ectopically expressed cells demonstrated slower GSH depletion under BSO treatment (Supplementary Fig. 4E). Thus, our results indicates that lncRNA GDIL induces intracellular accumulation by inhibiting GSH degradation.

### GSH accumulation induced by GDIL is required for platinum resistance

GSH plays its crucial role in chemoresistance as an antioxidant to buffer oxidative stress induced by anti-cancer drugs. We investigated whether increased GSH by GDIL upregulation contributed to ROS scavenging and cell survival under platinum treatment.

**Fig. 4:**
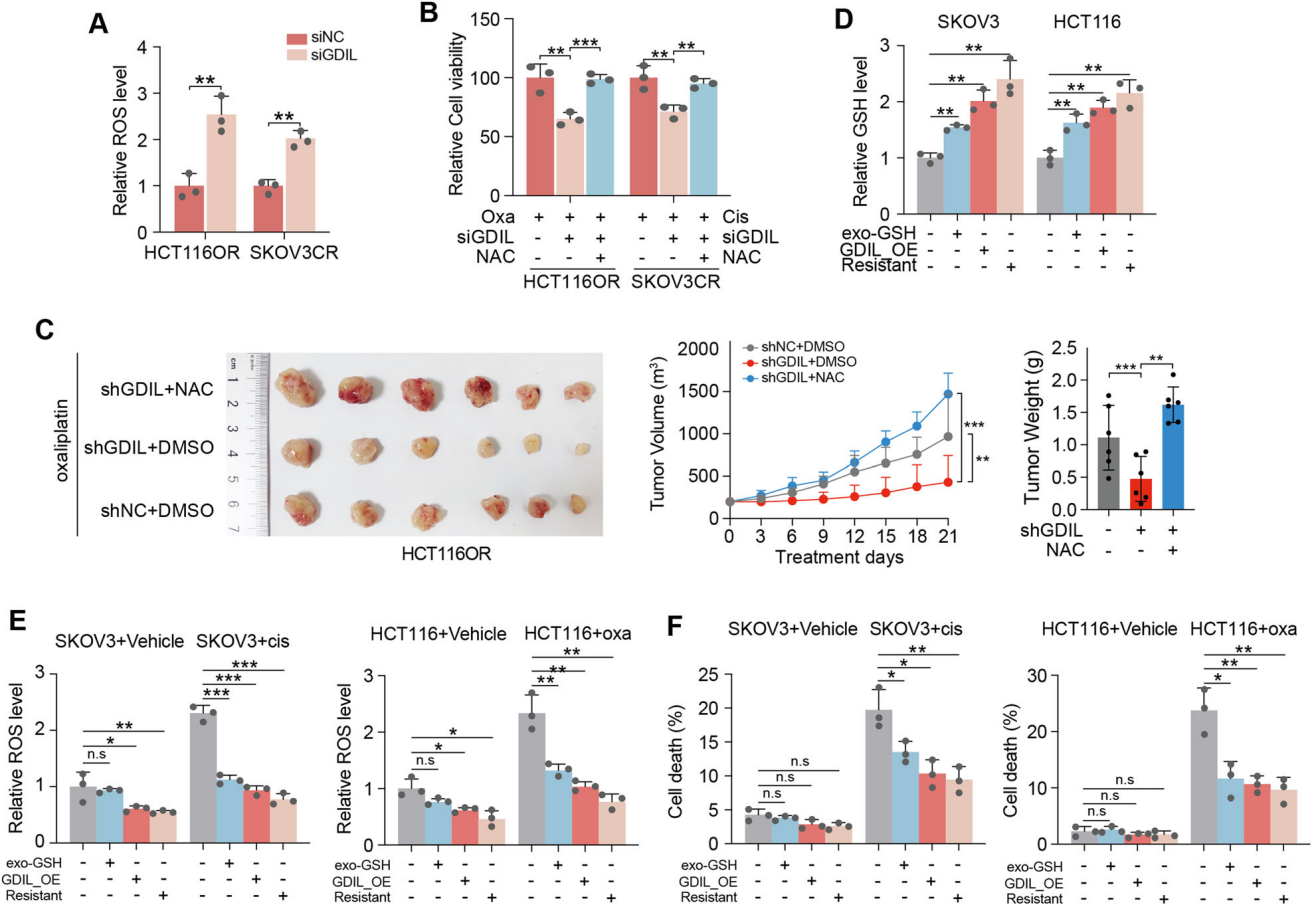
GSH accumulation induced by GDIL is required for platinum resistance. **A** Relative ROS levels in control and GDIL knockdown HCT116OR and SKOV3CR cells. **B** Cell viability of HCT116OR and SKOV3CR cells treated with GDIL targeting siRNAs alone or in combination with NAC (10 mM), exposed to 2 μM oxaliplatin or 2 μM cisplatin for 72 h. **C** Control (shNC) or GDIL knockdown (shGDIL) HCT116OR-CDXs treated with oxaliplatin (5 mg/kg) with or without NAC (5 g/liter NAC was supplemented in drinking water, pH 6.6 to 7.2) shown in representative images (left), tumor growth (middle) and weight change (right), *n* = 6 per group. **D**–**F** Relative GSH, ROS and cell death with indicated treatment, oxaliplatin: 0.5 μM, cisplatin: 0.5 μM.

It was demonstrated that ROS levels were increased by silencing GDIL (Fig. 4A). We utilized NAC to neutralize increased levels of ROS. This could rescue the decreased cell viability by GDIL knockdown (Fig. 4B). When H_2_O_2_ was added to induce ROS levels, cell viability was further diminished by GDIL knockdown (Supplementary Fig. 5A, B). These findings were recapitulated by xenograft models. GDIL knockdown resensitized an oxaliplatin-resistant HCT116 CDXs to therapy in vivo, however, these effects were blocked when combining treatment with NAC (Fig. 4C).

We then tested whether GDIL promoted GSH upregulation in platinum resistant cells is sufficient to block ROS induced cell death. Matched GSH levels, ROS levels and cell apoptosis were determined in 1) parental cells, 2) parental cells treated with exogenous GSH, 3) parental cells with GDIL overexpressed, 4) resistant cells. We showed that although supplementation of exogenous GSH only caused a slight recovery of intracellular GSH (GSH levels less than those in group 2 and 3), it could drastically deplete ROS and rescue cell death induced by platinum (Fig. 4D–F). These data indicate that GDIL, by maintaining high levels of GSH through preventing its degradation inside the cells, is sufficient to induce platinum resistance in a ROS-dependent manner.

### GDIL downregulates CHAC1 to inhibit GSH degradation

**Fig. 5:**
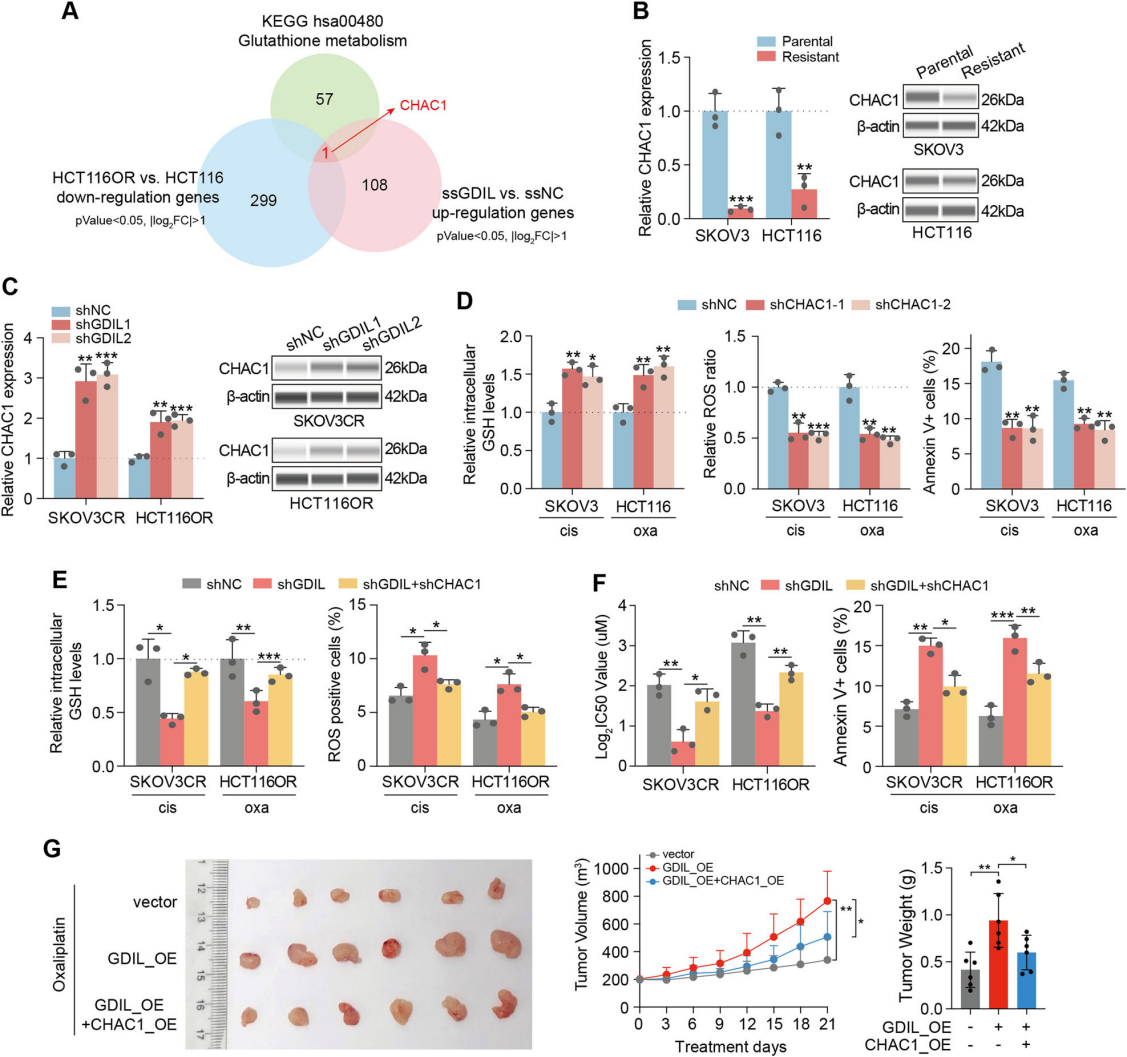
GDIL downregulates CHAC1 to inhibit GSH degradation. **A** Venn analysis was performed to identify genes involved in GSH metabolism, upregulated in HCT116OR cells and down-regulated by GDIL knockdown. **B** CHAC1 mRNA and protein in parental and resistant cells. **C** CHAC1 mRNA and protein in GDIL silencing and control cells. **D** Relative GSH (left), ROS (middle) and apoptosis levels (right) in CHAC1 knockdown or control SKOV3 and HCT116 cells under cisplatin (0.5 μM) and oxaliplatin (0.5 μM) treatment, respectively. **E** Relative GSH (left) and ROS (right) levels in control (shNC), GDIL knockdown (shGDIL), GDIL knockdown+CHAC1 knockdown (shGDIL + shCHAC1) SKOV3CR and HCT116OR cells under cisplatin (0.5 μM) or oxaliplatin (0.5 μM) treatment. **F** Cell viability and apoptosis were examined in control (shNC), GDIL knockdown (shGDIL), GDIL knockdown+CHAC1 knockdown (shGDIL+shCHAC1) SKOV3CR and HCT116OR cells. **G** Vector, GDIL overexpression (GDIL_OE) or GDIL and CHAC1 overexpression (GDIL_OE + CHAC1_OE) HCT116-CDX tumors treated with oxaliplatin in representative images (left), tumor growth (middle) and weight change (right), *n* = 6 per group.

In order to understand GDIL’s potential downstream targets and how collectively they contribute to GSH degradation and platinum resistance, RNA sequencing data of GDIL knockdown and control cells was analyzed (Fig. 3A). Our RNA-seq data was intersected with GSH metabolism gene set from KEGG (hsa00480). One potential downstream gene, CHAC1, was significantly down-regulated in resistant cells (compared with parental ones), and upregulated in GDIL knockdown resistant cells (compared with control) (Fig. 5A). QRT-PCR and western blotting validated what we observed from RNA-seq data (Fig. 5B and C, Supplementary Fig. 6A). These findings suggests that CHAC1 is a critical target of and negatively regulated by GDIL.

CHAC1 is a newly discovered endoplasmic reticulum inducible gene, involved in the γ-glutamyl cycle that can degrade GSH and promote cell apoptosis [15]. Silencing CHAC1 induced GSH upregulation, ROS depletion and inhibited drug-induced apoptosis (Supplementary Fig. 6B and Fig. 5D). Whereas CHAC1 overexpression promoted GSH depletion, ROS accumulation and promoted drug-induced apoptosis in resistant cells (Supplementary Fig. 6C, D). Rescue assyas confirmed GDIL’s regulation on GSH levels, ROS and cell apoptosis depended on CHAC1 expression (Fig. 5E, F, Supplementary Fig. 6E–G). CHAC1 upregulation also resensitized GDIL overexpressed HCT116-CDX models to platinum treatment in vivo. CDX models formed by GDIL overexpression HCT116 cells promoted tumor survial under oxaliplatin treatment, while further ectopic expression of CHAC1 alleviated tumor resistance (Fig. 5G). Altogether, our data were consistent with the hypothesis that CHAC1 is a major target of GDIL and is directly involved in GDIL-mediated inhibition of GSH degradation and platinum chemoresistance.

### GDIL binds to and mediates the degradation of CHAC1 transcript

**Fig. 6:**
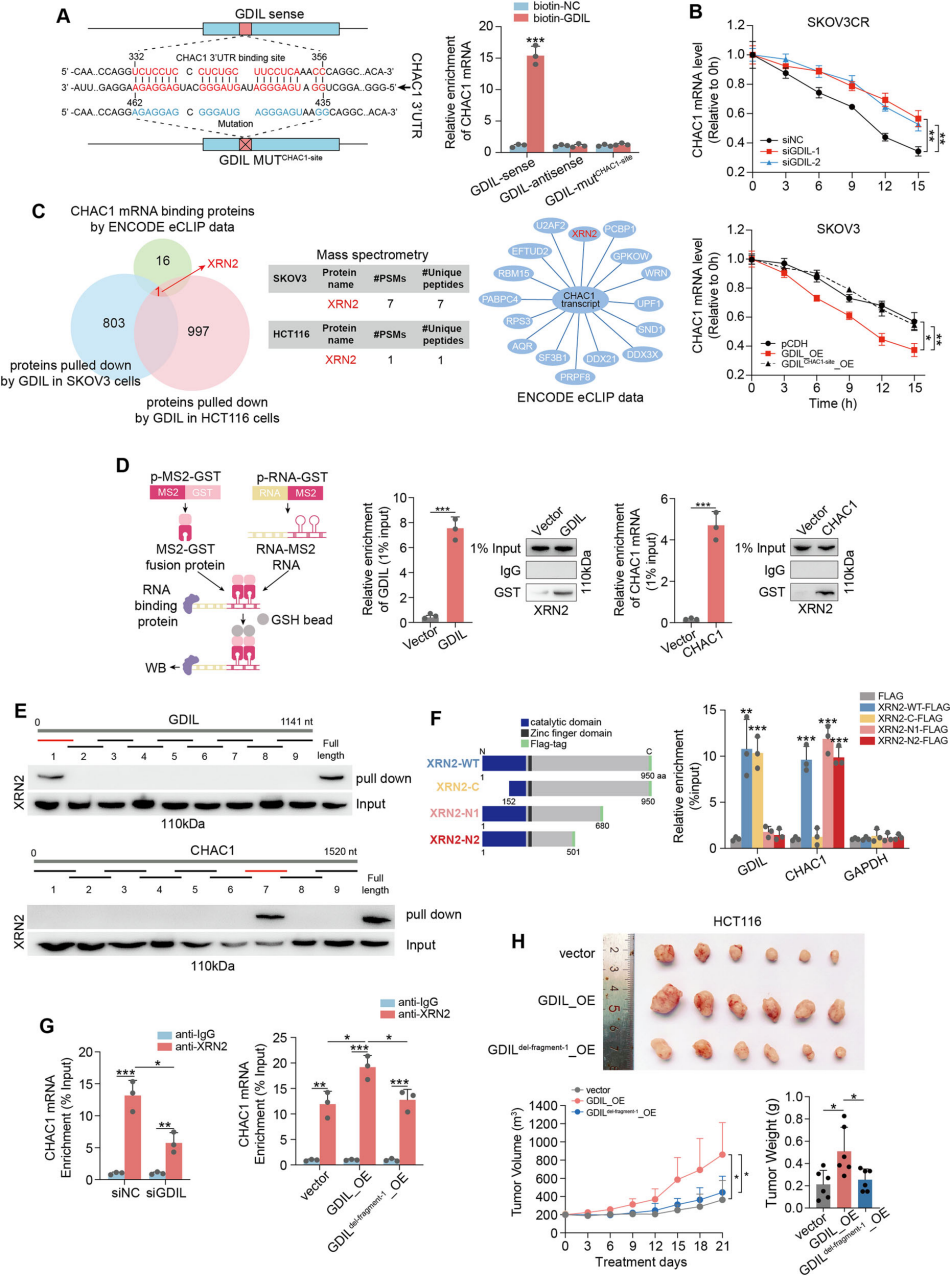
GDIL promotes CHAC1 mRNA degradation via XRN2. **A** Left: complementary sequence analysis between GDIL and CHAC1 mRNA; Right: CHAC1 mRNAs bind with sense, antisense and CHAC1 binding site mutated (MUT^CHAC1-site^) GDIL were examined by biotin pull-down assay and qRT-PCR. **B** The stability of CHAC1 mRNA in SKOV3CR (up) with GDIL knockdown, and SKOV3 (bottom) with wildtype or mutated GDIL overexpression was measured by qRT-PCR. RNA synthesis was blocked by α-amanitin, RNA levels were calculated relative to 0 h. **C** Venn analysis was performed to identify proteins pulled down by GDIL in both SKOV3 and HCT116 cells by our RNA pull down/Mass spectrometry analysis and also bind with CHAC1 mRNA from ENCODE eCLIP data. **D** MS2-pull down combined with qRT-PCR and immunoblotting showing interaction between XRN2 and GDIL. **E** RNA pull-down using GDIL and CHAC1 transcript segments. **F** RIP showing the interaction between XRN2 domains and GDIL, XRN2 domains and CHAC1 transcript. **G** Left: relative CHAC1 mRNA binding with XRN2 in control and GDIL silenced cells, represented as the percentage of the input bound; Right: relative CHAC1 mRNA binding with XRN2 in cells transfected with vector, overexpression of wild type and 1–200 nt (fragment 1) deleted GDIL, represented as the percentage of the input bound. **H** HCT116-CDXs transfected with vector, overexpression of wild type and 1–200 nt (fragment 1) deleted GDIL under oxaliplatin treatment shown in representative images (up), tumor growth (bottom left) and weight change (bottom right).

An important question remains how GDIL downregulates CHAC1. Toward this end, we analyzed the RNA sequences of GDIL and CHAC1 mRNA, and identified a 22 bp complementary region between them (Fig. 6A). The physical interaction between GDIL and CHAC1 mRNA was validated by affinity pull-down of endogenous CHAC1 transcript using in vitro transcribed, biotin-labeled GDIL. Results showed that CHAC1 mRNA were significantly enriched by sense GDIL but not by antisense or CHAC1-binding sequence mutated GDIL (Fig. 6A). GDIL-CHAC1 mRNA interaction was further validated by luciferase reporter assay (Supplementary Fig. 7A).

We previously showed that GDIL is mainly localized in the cytoplasm (see Supplementary Fig. 1F, G). We therefore examined whether cytoplasmic GDIL can bind to CHAC1 mRNA and regulate its stability. CRC cells were treated with α-amanitin to block RNA synthesis and CHAC1 mRNA degradation was tested over a 15 h period. Silencing GDIL delayed CHAC1 mRNA degradation (Fig. 6B, Supplementary Fig. 7B, C). By contrasct, GDIL overexpression accelerated CHAC1 mRNA degradation (Fig. 6B, Supplementary Fig. 7B, C). The half-life of CHAC1 mRNA was not affected by overexpression of GDIL with CHAC1-binding sequence mutation.

### GDIL promotes CHAC1 mRNA degradation via XRN2

So far, our results uncover degradation of CHAC1 transcript, mediated by GDIL through direct binding. Since lncRNA GDIL itself is not a ribonuclease, we next explore GDIL’s interacting protein in search of the mechanism by which GDIL mediates CHAC1 mRNA degradation. RNA pull-down and mass spectrometry identified in total 161 proteins specifically bind with sense GDIL compared with anti-sense one (Fig. 6C and Supplementary Table 5). Among them, XRN2 protein was also observed interact with CHAC1 mRNA by ENCODE eCLIP data (Fig. 6C). XRN2, a 5’-3’ exoribonuclease, has been shown to participate in mRNA decay [16]. We then confirmed the binding of GDIL with XRN2, and binding of CHAC1 mRNA with XRN2, by in vivo MS2 pull down/western blot assays and RIP (Fig. 6D and Supplementary Fig. 7D).

We further characterize XRN2/GDIL and XRN2/CHAC1 mRNA interaction. Series of biotin-labeled GDIL and CHAC1 fragments were constructed and their affinities with XRN2 were examined by immunoblotting. Fragment 1 containing 1–200 nt of GDIL and fragment 7 containing 950–1100 nt of CHAC1 mRNA were necessary for its interaction with XRN2 (Fig. 6E). Results from RIP assays using exogenously expressed shortened FLAG-XRN2 constructs showed that the carboxyl-terminal region and catalytic domain of XRN2 were responsible for binding to GDIL and CHAC1 mRNA, respectively (Fig. 6F). Our RIP results showed that physical association of CHAC1 mRNA with XRN2 decreased after GDIL knockdown (Fig. 6G). By constrast, CHAC1 mRNA enriched by XRN2 increased upon GDIL overexpression, whereas this effect was partially abolished by deletion of fragment 1 (1–200 nt) in GDIL (Fig. 6G). Additionally, the increased CHAC1 mRNA decay, GSH accumulation, ROS scavenging, drug resistant phenotypes in vitro and in vivo induced by GDIL overexpression was blocked when fragment 1 (1–200 nt) in GDIL was deleted (Supplementary Fig. 7E–G and Fig. 6H). Collectively, these results reveal that GDIL promotes CHAC1 mRNA degradation by recruiting XRN2 and forming triplexes with CHAC1 and XRN2.

### GDIL binds to XRN2 and induces its cytoplasmic translocation

**Fig. 7:**
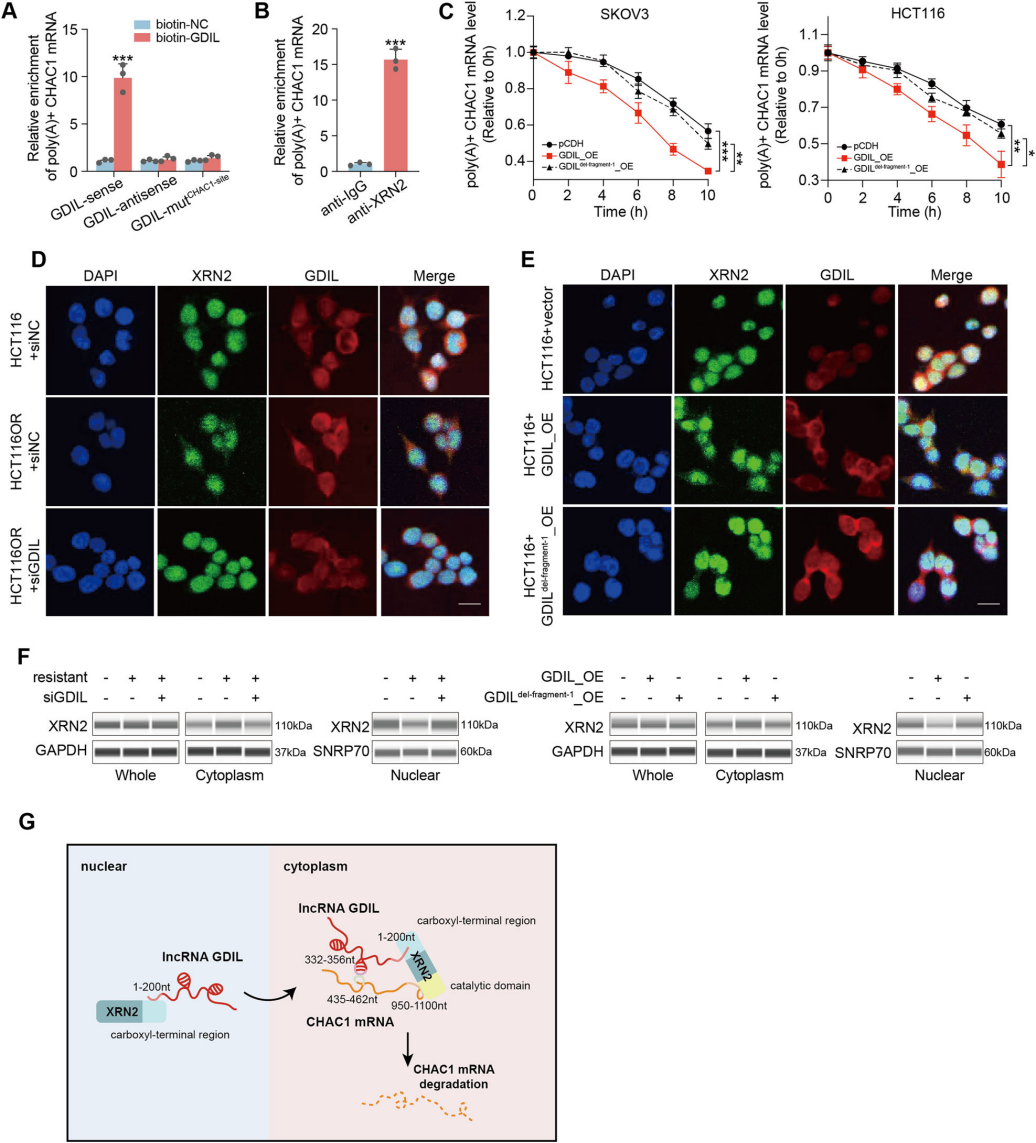
GDIL induces cytoplasmic translocation of XRN2. **A** Poly(A) + CHAC1 mRNA bind with sense, antisense and CHAC1 binding site mutated (MUT^CHAC1-site^) GDIL were examined by biotin pull-down assay and qRT-PCR. **B** Relative Poly(A) + CHAC1 mRNA binding using anti-XRN2 antibody or IgG in HCT116 cells, represented as the percentage of the input bound. **C** Stability of Poly(A) + CHAC1 mRNA in SKOV3 and HCT116 cells with control, overexpression of wildtype or fragment 1 deleted GDIL was measured by qRT-PCR. **D** RNA FISH for GDIL (red) and immunofluorescence (IF) for XRN2 (green) in parental and resistant HCT116 cells. Blue: DAPI; right: merge. Scale bars, 10 mm. **E** RNA FISH for GDIL (red) and immunofluorescence (IF) for XRN2 (green) in HCT116 cells with GDIL overexpressed. Blue: DAPI; right: merge. Scale bars, 10 mm. **F** Subcellular localization of XRN2 protein was detected by immunoblotting in HCT116 cells. SNRP70 was used as nuclear control. GAPDH was used as cytoplasmic control. **G** Schematic model of GDIL/XRN2/CHAC1 binding.

Mature mRNAs are exported from nucleus to cytoplasm where they are transcribed, spliced, capped and polyadenylated. Next, we purified poly(A)+ RNAs and proved the binding of mature processed CHAC1 mRNA with GDIL and XRN2, respectively (Fig. 7A, B). Further, decay of poly(A) + CHAC1 mRNA was accelerated by GDIL overexpression, while this effect was blocked when deletion of fragment 1 (1-200nt) in GDIL (Fig. 7C). These results suggested that, GDIL contributes to cytoplasm located mature CHAC1 mRNA degradation via XRN2. Although XRN2 was reported predominantly localized in the nucleus, it was discovered in recent studies as a compositional protein of stress granules (SGs) located in the cytoplasm [16–18]. Since the expression level of XRN2 is not dramatically regulated by GDIL (Supplementary Fig. 8A), we wondered whether GDIL is required for XRN2’s localization into cytoplasm. To experimentally validate our hypothesis, we performed RNA fluorescence in situ hybridization (FISH) and immunofluorescence (IF) staining for GDIL and XRN2, respectively, in parental and resistant conditions. In parental CRC cells, XRN2 was predominantly localized to the nucleus, whereas in resistant cells, a fraction of XRN2 is colocalized to GDIL-positive loci in cytoplasm (Fig. 7D). Absence of GDIL via siRNA failed to re-localize XRN2 from nuclear to cytoplasm in chemo-resistant cells. By contrast, GDIL overexpression enhance cytoplasmic translocation of XRN2 in parental CRC cells, while this effect could be blocked when overexpressed GDIL with fragment 1 (1–200 nt) deleted (Fig. 7E). These observations were further validated by immunoblotting assays (Fig. 7F). Overall, these detailed analyses highlight that GDIL assists the translocation of XRN2 from nuclear to cytoplasm so to bind and degrade CHAC1 mRNA (Fig. 7G).

### Therapeutic targeting of GDIL delays platinum resistance

**Fig. 8:**
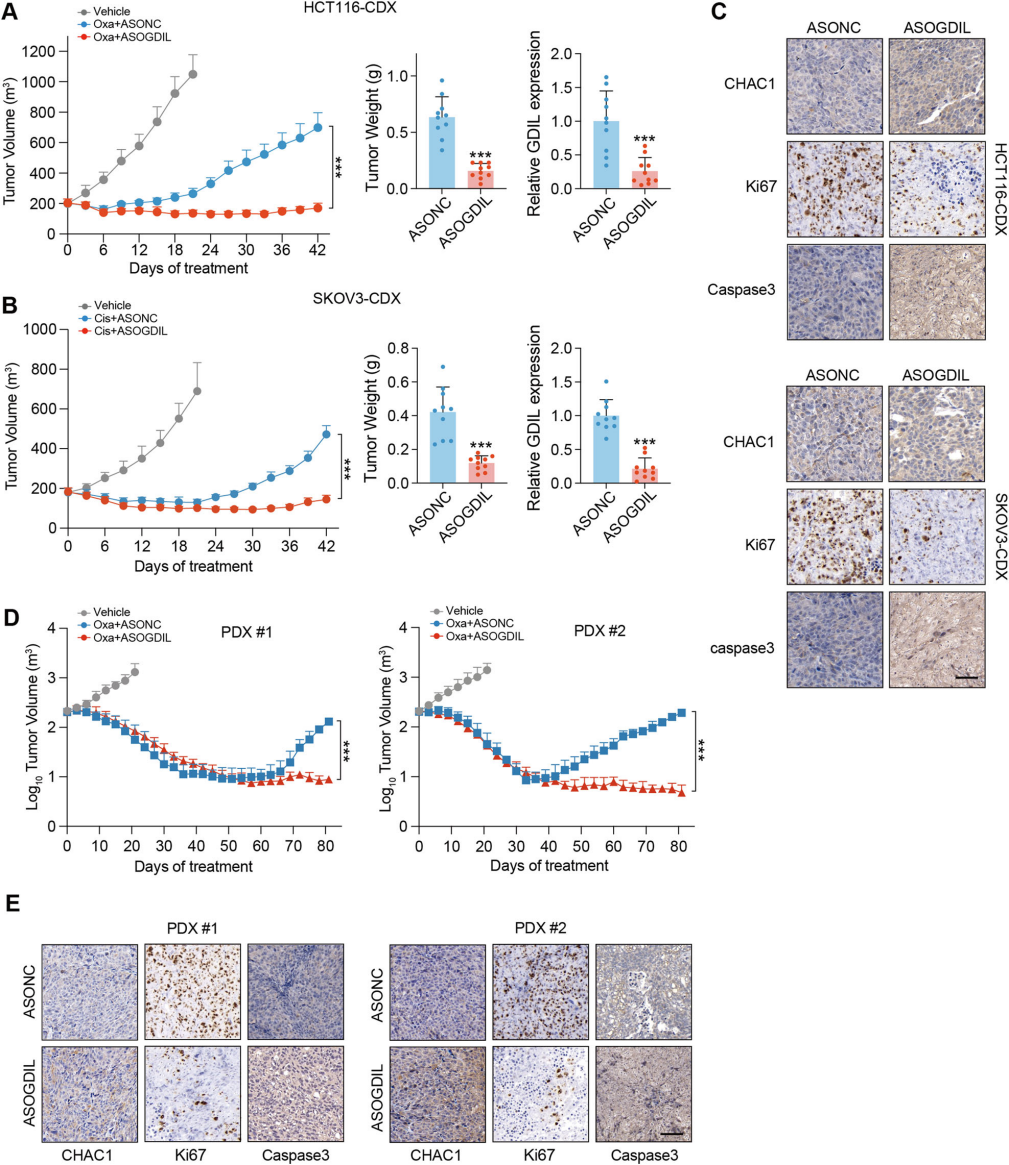
Therapeutic targeting of GDIL delays platinum resistance. **A**, **B** Targeting GDIL in HCT116-CDX (**A**) and in SKOV3-CDX (**B**). Growth of tumors over time (left), weights of tumors (middle) and GDIL levels in tumors (right) at the end of experiment are shown. Oxaliplatin (5 mg/kg, every 3 days) for HCT116-CDX and cisplatin (3 mg/kg, daily) for SKOV3-CDX was administered through intraperitoneal injection. *n* = 10. **C** CDX tumors IHC staining. Scale bar, 100 μm. **D** Tumor volumes of PDXs over time. Oxaliplatin (5 mg/kg, every 3 days) was administered through intraperitoneal injection. ASOs were administered via subcutaneous peritumor injection (50 mg/kg, every 3 days). *n* = 6. **E** qRT-PCR of GDIL and IHC staining of CHAC1, ki-67 and caspase 3 in PDX tumors. Scale bar, 50 μm.

Our data demonstrated the role of GDIL in inhibiting GSH degradation and inducing platinum resistance. These findings indicated the potential value of GDIL as a target to reverse resistance. We therefore examined whether targeting of GDIL by specific locked nucleic acid (LNA)-modified ASOs, could delay resistance to platinum by various models. HCT116-CDXs initially responded to platinum with growth retardation and volume reduction. After continuous platinum administration, HCT116-CDXs gradually acquired resistance, as seen by lost sensitivity and tumor relapse (Fig. 8A). Combination of oxaliplatin and LNA-ASO could silenced GDIL in CDX and delayed tumor relapse (Fig. 8A). We also implanted SKOV3 subcutaneously to construct SKOV3-CDX mouse models. Continuously explosion under platinum also induce resistance, whereas combination treatment resensitize tumors to platinum, without demonstrating body weight loss in mice (Fig. 8B and Supplementary Fig. 8B). The combination effect of GDIL knockdown with platinum was recapitulated in immunohistochemistry (IHC) staining analysis of the xenograft tumors, where GDIL-ASO enhanced the inhibition effect of platinum on tumor cell proliferation markerd by Ki-67 staining and apoptosis marked by caspase 3 (Fig. 8C). Consistently, CHAC1 protein was upregulated by GDIL knockdown (Fig. 8B, C).

The effect of GDIL silencing on delaying resistance to platinum was confirmed in patient-derived xenograft (PDX) models. PDXs are more clinically relevant and predictive compared with CDXs, making them irreplaceable choice in cancer treatment studies [19]. We collected fresh cancer tissues from 2 CRC patients and PDXs were established. Both PDXs were initially responded to oxaliplatin, but over time developed resistance after ongoing treatment (Fig. 8D). Combination of GDIL-ASO and oxaliplatin efficiently inhibited acquired resistance (Fig. 8D). Furthermore, this combination treatment did not induce reduction of body weight in mice (Supplementary Fig. 8C). Consistent with CDXs, IHC staining showed that PDXs exposed to GDIL-ASO induced CHAC1 and caspase 3 expression and decreased ki-67 levels, compared with oxaliplatin therapy alone (Fig. 8E). Thus, we comfirmed that GDIL is a potential target to delay acquired platinum resistance in CDX and PDX mouse models.

## Discussion

Platinum-based agents, including cisplatin, carboplatin and oxaliplatin, are extensively used in chemotherapy against ovarian, colorectal, breast and bladder cancers etc. However, drug resistance greatly restricts clinical application of platinum and the underlying mechanisms are intricate. In addition to impact DNA structure and function, the principal actin mechanism of platinum is to generate ROS to lead to cell death [20, 21]. By preventing excessive ROS, antioxidants including GSH plays an important role in enabling cancer cell survival [22]. Enhanced GSH accumulation and elevated apoptosis threshold were reported to have a profound influence on platinum resistance [22, 23]. Developing strategies to target these key participators would help to increase patient eligibility for platinums as well as make these treatments more effective.

In search for critical regulators for platinum resistance, we performed transcriptomic analysis and siRNA screening in resistant and parental CRC cells. Among the candidate transcripts, lncRNA GDIL was shown to upregulate in resistant cell lines and most drastically affect drug response to oxaliplatin. Consistently, expression of GDIL in recurrent CRC tissues after oxaliplatin-based therapy were >10 folds than pre-treated tissues. Increased resistance was also induced by GDIL under cisplatin and carboplatin treatment in ovarian cancer cell line and xenograft mouse models. Thus, we identified a lncRNA molecule that drives resistance to various generations of platinum.

The maintenance of intracellular GSH homeostasis involves both synthesis and degradation mechanism [14]. However, little is known about whether regulation of GSH degradation is involved in platinum-resistant cancers. Our metabolomic and metabolic flux analysis reveals that GSH metabolism is one of the top pathways regulated by GDIL. GDIL promotes GSH accumulation, ROS scavenging and cell survival. This is attributed to decreased GSH degradation.

GDIL is a noncoding RNA containing 114nt identified by our RACE assay. It mainly locates in the cytoplasm of cancer cells. We profiled the downstream target of GDIL and demonstrated that CHAC1 was a key target downregulated by GDIL. As a γ-glutamylcyclotransferase, CHAC1 has been shown to specifically degrade intracellular GSH into 5-oxoproline and cysteine-glycine to control the intracellular redox milieu [15]. Recently, CHAC1 was also discovered to be a key gene in ferroptosis and to augment cancer immunotherapy [24]. However, the contribution of CHAC1 to platinum resistance in cancer has not been studied.

Basal levels of CHAC1 are tightly suppressed in cancer cells [24]. A sophisticated mechanism for regulating CHAC1 expression is through transcriptional activation by ATF4 [15, 25], but the understanding of how lncRNAs manipulate CHAC1 expression is limited. LncRNAs enriched in the cytoplasm have been reported to regulate mRNA stability, representing one of the major ways of controlling expression of genetic information [26]. Our study showed that interaction with GDIL reduced CHAC1 mRNA stability and, subsequently, CHAC1 expression. Mechanistically, we identified GDIL as a scaffold to enhance specific interactions between XRN2 protein and CHAC1 mRNA.

**Fig. 9:**
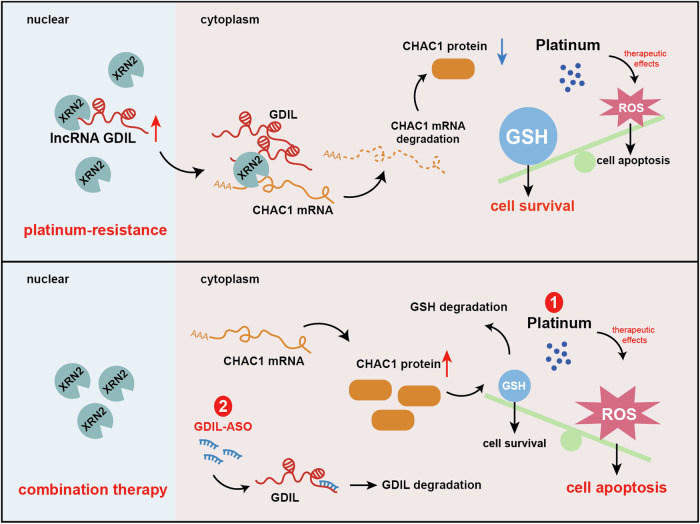
Schematic model of this study. Top: regulation of GDIL-glutathione degradation axis by XRN2/CHAC1 in platinum resistant cancer cells. Bottom: combination therapeutic strategy against platinum resistance by adding ASO targeting lncRNA GDIL.

RNA degradation can occur in both the nuclear and cytosolic compartments at different life stages of RNA transcripts. Mature mRNAs are exported to the cytoplasm from the nucleus after they are transcribed, spliced, capped, and polyadenylated. We verified the binding of mature processed CHAC1 mRNA with GDIL and XRN2 by purification of poly(A)+ RNAs that were pulled down. These results indicated that CHAC1 mRNA degradation by GDIL/XRN2 most likely occurred in the cytoplasm. A major issue that needs to be solved is, as a nuclear-enriched 5′ to 3′ exoribonuclease, how does XRN2 function to decay CHAC1 mRNA in the cytoplasm. Nucleo-cytoplasmic shuttling is a common property of many RNA-binding proteins. These processes are also under the control of lncRNAs. For example, our previous work found that lncRNA CCAL, by interacting with HuR and facilitating its translocation to the cytoplasm, activated the β-catenin pathway and chemoresistance in CRC [27]. Viegas et al. recently reported that XRN2 could translocate to cytosolic small RNA granules to function [16]. Therefore, we hypothesized and validated the mechanism by which GDIL not only interacted with XRN2, but also promoted its re-localization from the nucleus to the cytoplasm, where XRN2 could meet CHAC1 mRNA and further degrade it (Fig. 9).

Once in the cytoplasm, the 5’ cap could recruit protein complexes that protect mRNA from decapping enzymes and ribonucleases. It is known that 5’-3’ exonuclease requires decapping of target transcript before it could function [28]. A decapping factor(s) is expected to be involved in mRNA decay of CHAC1 aside from XRN2. Among GDIL binding proteins from our RNA pull-down and mass spectrometry data, we have not identified such a factor with decapping function. A possible explanation is that the interaction between decapping factors and target mRNAs is transient [29]. Further study is warranted to increase our understanding of this process.

Interfering GSH synthesis has been applied for cancer treatment, although with poor clinical response. Strategies targeting GSH degradation pathways have not yet been developed. We identified GDIL as a crucial inhibitor of GSH degradation and inducer of platinum resistance, making it an ideal target for reversing cancer recurrence. ASO agents for RNA-based therapeutics have been approved by the US Food and Drug Administration (FDA) and European Medicines Agency (EMA) [30]. This new class of drugs is sparking hope for therapy for human diseases, including cancer. Therapeutic targeting of GDIL was developed for platinum-resistant CRC patients, and our results suggest that the combination of oxaliplatin and GDIL ASO could largely suppress tumor recurrence in CDX and PDX models. This combination regimen deserves further validation in clinical trials and may exert positive effects on the prognosis of platinum-resistant patients.

Collectively, we have shown that lncRNA GDIL is a promising prognostic indicator for platinum resistance. Our work also demonstrates that GDIL promotes intracellular accumulation and targeted inhibition of GDIL resensitizes chemoresistance of CDX and PDX models. Therefore, it might be promising to target GDIL to improve the antitumor efficacy of platinum-based chemotherapy.

## Supplementary information

Supplementary Figures

Supplementary Figures-uncropped original blots

Supplementary Tables

Supplementary Materials and Methods

## Data Availability

The data that support the findings of this study are available from the corresponding author upon reasonable request.

## References

[1] Forman HJ, Zhang H. Targeting oxidative stress in disease: promise and limitations of antioxidant therapy. Nat Rev Drug Discov. 2021;20:689–709. 34194012 10.1038/s41573-021-00233-1 PMC8243062

[2] Birben E, Sahiner UM, Sackesen C, Erzurum S, Kalayci O. Oxidative stress and antioxidant defense. World Allergy Organ J. 2012;5:9–19. 23268465 10.1097/WOX.0b013e3182439613 PMC3488923

[3] Bansal A, Simon MC. Glutathione metabolism in cancer progression and treatment resistance. J Cell Biol. 2018;217:2291–8. 29915025 10.1083/jcb.201804161 PMC6028537

[4] Arrick BA, Nathan CF, Griffith OW, Cohn ZA. Glutathione depletion sensitizes tumor cells to oxidative cytolysis. J Biol Chem. 1982;257:1231–7. 6799503

[5] Harris IS, Treloar AE, Inoue S, Sasaki M, Gorrini C, Lee KC, et al. Glutathione and thioredoxin antioxidant pathways synergize to drive cancer initiation and progression. Cancer Cell. 2015;27:211–22. 25620030 10.1016/j.ccell.2014.11.019

[6] Harris IS, Endress JE, Coloff JL, Selfors LM, McBrayer SK, Rosenbluth JM, et al. Deubiquitinases maintain protein homeostasis and survival of cancer cells upon glutathione depletion. Cell Metab. 2019;29:1166–81.e6. 30799286 10.1016/j.cmet.2019.01.020 PMC6506399

[7] Estrela JM, Ortega A, Obrador E. Glutathione in cancer biology and therapy. Crit Rev Clin Lab Sci. 2006;43:143–81. 16517421 10.1080/10408360500523878

[8] Giustarini D, Milzani A, Dalle-Donne I, Rossi R. How to increase cellular glutathione. Antioxidants (Basel). 2023;12:1094. 10.3390/antiox12051094 PMC10215789 37237960

[9] Deng X, Li S, Kong F, Ruan H, Xu X, Zhang X, et al. Long noncoding RNA PiHL regulates p53 protein stability through GRWD1/RPL11/MDM2 axis in colorectal cancer. Theranostics. 2020;10:265–80. 31903119 10.7150/thno.36045 PMC6929633

[10] Deng X, Kong F, Li S, Jiang H, Dong L, Xu X, et al. A KLF4/PiHL/EZH2/HMGA2 regulatory axis and its function in promoting oxaliplatin-resistance of colorectal cancer. Cell Death Dis. 2021;12:485. 33986248 10.1038/s41419-021-03753-1 PMC8119946

[11] Huan L, Guo T, Wu Y, Xu L, Huang S, Xu Y, et al. Hypoxia induced LUCAT1/PTBP1 axis modulates cancer cell viability and chemotherapy response. Mol Cancer. 2020;19:11. 31964396 10.1186/s12943-019-1122-z PMC6971890

[12] Mansoori B, Mohammadi A, Davudian S, Shirjang S, Baradaran B. The different mechanisms of cancer drug resistance: a brief review. Adv Pharm Bull. 2017;7:339–48. 29071215 10.15171/apb.2017.041 PMC5651054

[13] Chen X, Chen S, Yu D. Metabolic Reprogramming of chemoresistant cancer cells and the potential significance of metabolic regulation in the reversal of cancer chemoresistance. Metabolites. 2020;10:289. 10.3390/metabo10070289 PMC7408410 32708822

[14] Baudouin-Cornu P, Lagniel G, Kumar C, Huang ME, Labarre J. Glutathione degradation is a key determinant of glutathione homeostasis. J Biol Chem. 2012;287:4552–61. 22170048 10.1074/jbc.M111.315705 PMC3281641

[15] Crawford RR, Prescott ET, Sylvester CF, Higdon AN, Shan J, Kilberg MS, et al. Human CHAC1 protein degrades glutathione, and mRNA induction is regulated by the transcription factors ATF4 and ATF3 and a bipartite ATF/CRE regulatory element. J Biol Chem. 2015;290:15878–91. 25931127 10.1074/jbc.M114.635144 PMC4505494

[16] Viegas JO, Azad GK, Lv Y, Fishman L, Paltiel T, Pattabiraman S, et al. RNA degradation eliminates developmental transcripts during murine embryonic stem cell differentiation via CAPRIN1-XRN2. Dev Cell. 2022;57:2731–44.e5. 36495875 10.1016/j.devcel.2022.11.014 PMC9796812

[17] Marmor-Kollet H, Siany A, Kedersha N, Knafo N, Rivkin N, Danino YM, et al. Spatiotemporal proteomic analysis of stress granule disassembly using APEX reveals regulation by SUMOylation and links to ALS pathogenesis. Mol Cell. 2020;80:876–91.e6. 33217318 10.1016/j.molcel.2020.10.032 PMC7816607

[18] Markmiller S, Soltanieh S, Server KL, Mak R, Jin W, Fang MY, et al. Context-dependent and disease-specific diversity in protein interactions within stress granules. Cell. 2018;172:590–604.e13. 29373831 10.1016/j.cell.2017.12.032 PMC5969999

[19] Zanella ER, Grassi E, Trusolino L. Towards precision oncology with patient-derived xenografts. Nat Rev Clin Oncol. 2022;19:719–32. 36151307 10.1038/s41571-022-00682-6

[20] Zhang C, Xu C, Gao X, Yao Q. Platinum-based drugs for cancer therapy and anti-tumor strategies. Theranostics. 2022;12:2115–32. 35265202 10.7150/thno.69424 PMC8899578

[21] Perillo B, Di Donato M, Pezone A, Di Zazzo E, Giovannelli P, Galasso G, et al. ROS in cancer therapy: the bright side of the moon. Exp Mol Med. 2020;52:192–203. 32060354 10.1038/s12276-020-0384-2 PMC7062874

[22] Asantewaa G, Harris IS. Glutathione and its precursors in cancer. Curr Opin Biotechnol. 2021;68:292–9. 33819793 10.1016/j.copbio.2021.03.001

[23] Zhou J, Kang Y, Chen L, Wang H, Liu J, Zeng S, et al. The drug-resistance mechanisms of five platinum-based antitumor agents. Front Pharmacol. 2020;11:343. 32265714 10.3389/fphar.2020.00343 PMC7100275

[24] Xue Y, Lu F, Chang Z, Li J, Gao Y, Zhou J, et al. Intermittent dietary methionine deprivation facilitates tumoral ferroptosis and synergizes with checkpoint blockade. Nat Commun. 2023;14:4758. 37553341 10.1038/s41467-023-40518-0 PMC10409767

[25] Hamano M, Tomonaga S, Osaki Y, Oda H, Kato H, Furuya S. Transcriptional activation of Chac1 and other Atf4-target genes induced by extracellular l-serine depletion is negated with glycine consumption in Hepa1-6 hepatocarcinoma cells. Nutrients. 2020;12:3018. 10.3390/nu12103018 PMC7600170 33023086

[26] Carlevaro-Fita J, Johnson R. Global positioning sytem: understanding long noncoding RNAs through subcellular localization. Mol Cell. 2019;73:869–83. 30849394 10.1016/j.molcel.2019.02.008

[27] Deng X, Ruan H, Zhang X, Xu X, Zhu Y, Peng H, et al. Long noncoding RNA CCAL transferred from fibroblasts by exosomes promotes chemoresistance of colorectal cancer cells. Int J Cancer. 2020;146:1700–16. 31381140 10.1002/ijc.32608

[28] Brannan K, Kim H, Erickson B, Glover-Cutter K, Kim S, Fong N, et al. mRNA decapping factors and the exonuclease Xrn2 function in widespread premature termination of RNA polymerase II transcription. Mol Cell. 2012;46:311–24. 22483619 10.1016/j.molcel.2012.03.006 PMC3806456

[29] Wojtas MN, Pandey RR, Mendel M, Homolka D, Sachidanandam R, Pillai RS. Regulation of m(6)A transcripts by the 3’→5’ RNA elicase YTHDC2 is essential for a successful meiotic program in the mammalian germline. Mol Cell. 2017;68:374–87.e12. 29033321 10.1016/j.molcel.2017.09.021

[30] Winkle M, El-Daly SM, Fabbri M, Calin GA. Noncoding RNA therapeutics - challenges and potential solutions. Nat Rev Drug Discov. 2021;20:629–51. 34145432 10.1038/s41573-021-00219-z PMC8212082

