# Supplementary material for: Long noncoding RNA GDIL acts as a scaffold for CHAC1 and XRN2 to promote platinum resistance of colorectal cancer through inhibition of glutathione degradation
Source: Cell Death Dis. 2025 Feb 1;16(1):62. doi: 10.1038/s41419-025-07374-w (PMC11787370; doi:10.1038/s41419-025-07374-w)

**Supplementary Figure Legends:**

**Supplementary Figure 1. Characteristics of lncRNA GDIL. A** Relative expression of 7 genes in parental and oxaliplatin resistant C4 and R21 cells. **B** Relative cell viability in oxaliplatin resistant (OR) SW480 and HCT116 cells with each of the 7 candidate genes knocked down. **C** Representative image of PCR products from the 5’-RACE, 5’-nested RACE, 3’-RACE and 3’- nested RACE of GDIL are shown (left). The full-length 1141 nt GDIL sequence is shown on the right. **D** Protein coding potential of GDIL analyzed by ORF finder software from the National Center for Biotechnology Information (NCBI, https://www.ncbi.nlm.nih.gov/orffinder/). **E** Protein coding potential of GDIL analyzed by PhyloCSF software. GAPDH and β-actin served as the positive controls of coding genes, and MALAT1 served as the negative control of non-coding genes. **F** Expression of GDIL in cytoplasmic and nuclear fractionations of SKOV3, OVCAR3, HCT116 and SW480 cells. U6 RNA serves as a positive control for nuclear gene expression and GAPDH as a positive control for cytoplasmic gene expression. **G** RNAscope staining of GDIL in parental, cisplatin resistant SKOV3 (SKOV3CR) and oxaliplatin resistant HCT116 (HCT116OR) cells was performed. Representative images are shown. Scale bars: 5 μm. **H** The RNA copy numbers of GDIL were measured.

**Supplementary Figure 2. GDIL dependence in oxaliplatin-resistant CRC. A** Knockdown efficiency of siRNAs and ASO targeting GDIL were determined by qRT-PCR. **B, C** Relative cell viability (**B**) and growth (**C**) in parental (Par) and oxaliplatin resistant (OR) SW480 and HCT116 cells with GDIL knockdown by ASO. **D** Relative cell viability of SW480 and HCT116 cells under oxaliplatin treatment (1μM and 2.5μM, respectively). GDIL expression was silenced by siRNAs or ASO. **E** Relative cell growth in parental (Par) and oxaliplatin resistant (OR) SW480 and HCT116 cells with GDIL knockdown under oxaliplatin treatment (12μM and 25μM, respectively).

**Supplementary Figure 3. GDIL induces resistance to multiple generations of platinum.** **A** Relative expression of GDIL in control (vector) and GDIL overexpression (GDIL_OE) cells were determined by qRT-PCR. **B** Relative cell viability of SKOV3 and OVCAR3 cells under cisplatin (2μM and 25μM, respectively) and carboplatin treatment (200μM and 60μM, respectively). **C** Control and overexpression of GDIL in SKOV3-CDX treated with cisplatin shown in tumor growth (left) and weight change (right). **D** Relative GDIL expression in CDXs tissues from **C**. **E** Relative GDIL expression in SKOV3 cells treated with indicated concentrations of cisplatin.

**Supplementary Figure 4. GDIL inhibits intracellular GSH degradation. A** Knockdown efficiency of smart silencer targeting GDIL was examined by qRT-PCR. **B** Heatmap showing differently expressed mRNA between GDIL knockdown or controls by RNA-seq analysis. Values are scaled as indicated (-2 to 2) (n = 3). **B** Total glutathione, GSH and GSSG in HCT116OR-CDX mice with GDIL knockdown (shGDIL) or control (shNC) treatment. GSH and GSSG were determined by LC-MS/MS analysis. The abundance of tumor GSH and GSSG was normalized by the tumor weight. **C** Relative metabolites abundance in GSH metabolism pathway in control (vector) and GDIL overexpression (GDIL_OE) HCT116 and SKOV3 cells, n=4 per group. **D** GSH synthesis pathway in GDIL activation versus control HCT116 cells. Isotype tracing was performed using 3,3′-^13^C_2_-cystine, n = 4 per group. **E** Total glutathione content in control (vector) and GDIL overexpression (GDIL_OE) SKOV3 and HCT116 cells exposed to 0-10^4^ nM BSO for 72 hours.

**Supplementary Figure 5. GSH accumulation induced by GDIL is required for platinum resistance. A** Relative ROS levels in cisplatin resistant SKOV3 (SKOV3CR) and oxaliplatin resistant HCT116 (HCT116OR) cells treated with GDIL targeting siRNAs alone or in combination with H_2_O_2_. **B** Relative cell viability in SKOV3CR and HCT116OR cells under indicated treatments.

**Supplementary Figure 6. GDIL downregulates CHAC1 to inhibit GSH degradation.** **A** Expression of CHAC1 mRNA and protein in GDIL overexpression or control cells analyzed by qRT-PCR and western blot. **B, C** Knockdown (**B**) and overexpression (**C**) efficiencies of CHAC1 was examined by qRT-PCR and western blot. **D** Relative GSH (left), ROS (middle) and apoptosis levels (right) in CHAC1 overexpression or control SKOV3CR and HCT116OR cells under cisplatin and oxaliplatin treatment, respectively. **E** Relative GSH (left) and ROS (right) levels in control (vector), GDIL overexpression (GDIL_OE), GDIL overexpression + CHAC1 overexpression (GDIL_OE+CHAC1_OE) SKOV3 and HCT116 cells. **F** Cell viability and apoptosis were examined in control (vector), GDIL overexpression (GDIL_OE), GDIL overexpression + CHAC1 overexpression (GDIL_OE+CHAC1_OE) SKOV3 and HCT116 cells. **G** GDIL levels were examined in CHAC1 knockdown (left) and CHAC1 overexpression (right) CRC cells.

**Supplementary Figure 7. GDIL promotes CHAC1 mRNA degradation via XRN2. A** Dual Luciferase Reporter Assay used to detect the relative luciferase activity in HEK293T cells co-transfected with siGDIL/siNC, wild type (WT) or GDIL binding site mutated (MUT^GDIL-site^) 3′-UTR of CHAC1. **B** Overexpression efficiency of wild type and CHAC1 binding site mutated GDIL in corresponding cells. **C** Stability of CHAC1 mRNA in HCT116OR (up) with GDIL knockdown, and HCT116 (bottom) cells with wildtype or mutated GDIL overexpression was measured by qRT-PCR relative to 0 h after blocking new RNA synthesis with α-amanitin. **D** Relative GDIL (left) and CHAC1 mRNA (right) binding using anti-XRN2 antibody or IgG in HCT116 cells, represented as the percentage of the input bound. **E** Overexpression efficiency of wild type and fragment 1 deleted GDIL in corresponding cells. **F** Stability of CHAC1 mRNA in SKOV3 and HCT116 cells with indicated treatment was measured by qRT-PCR relative to 0 h after blocking new RNA synthesis with α-amanitin. **G** Relative GSH (left), ROS (middle) and apoptosis levels (right) in SKOV3 and HCT116 cells under cisplatin and oxaliplatin treatment, respectively.

**Supplementary Figure 8. Regulation of GDIL on XRN2 expression. A** XRN2 levels in GDIL overexpression cells were examined by qRT-PCR and western blot. **B** The effect of the combination of GDIL-ASO with oxaliplatin (left) or cisplatin (right) on mouse body weight loss in HCT116-CDX (left) and SKOV3-CDX (right). The body weights of CDX-bearing mice were monitored every 3 days. N=6 per group. **C** Combination of GDIL-ASO with oxaliplatin effect on mouse body weight loss. The body weights of PDX-bearing mice were monitored every 3 days. N=6 per group.


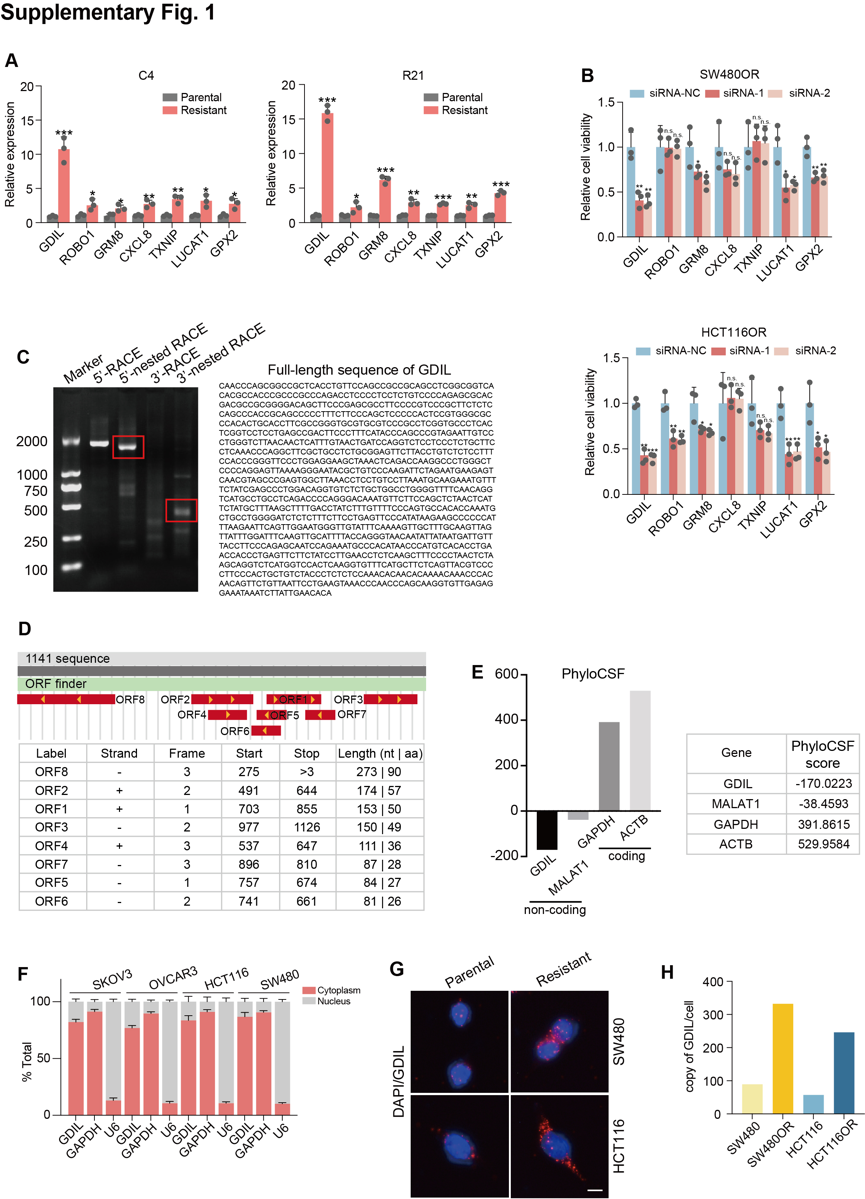


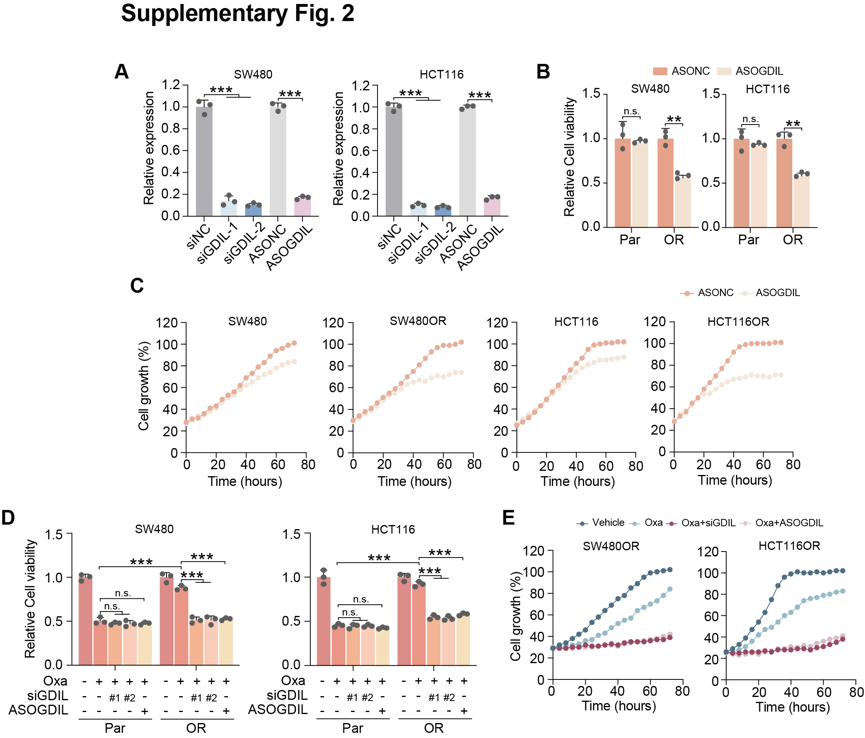


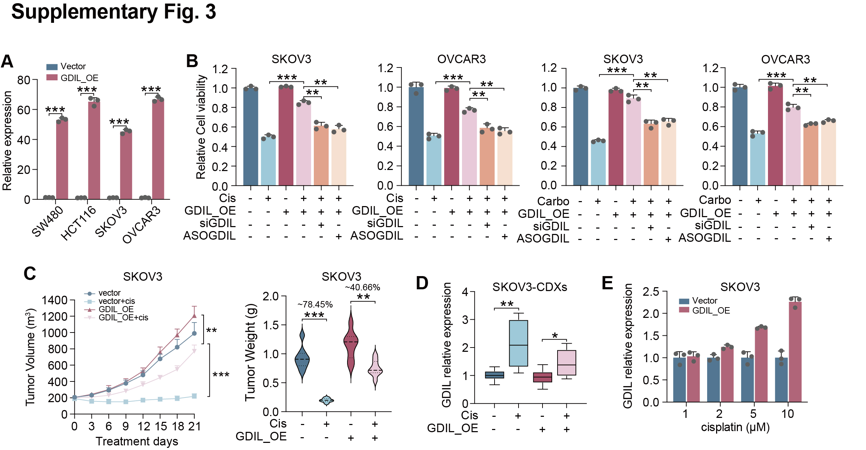


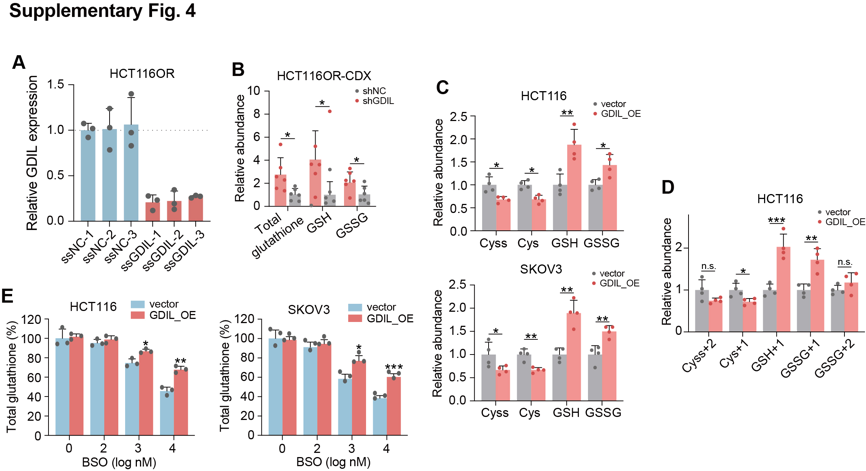


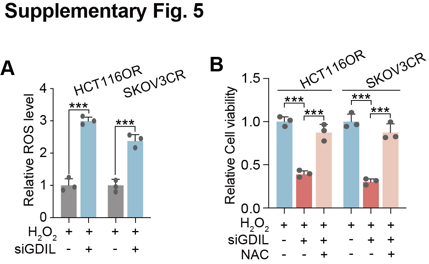


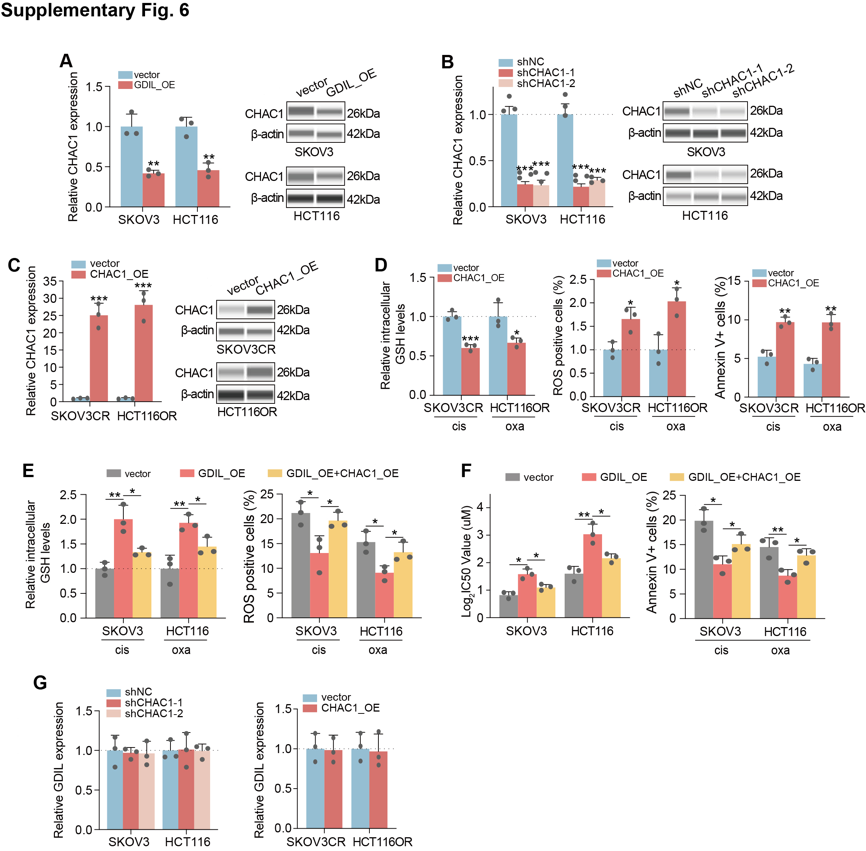


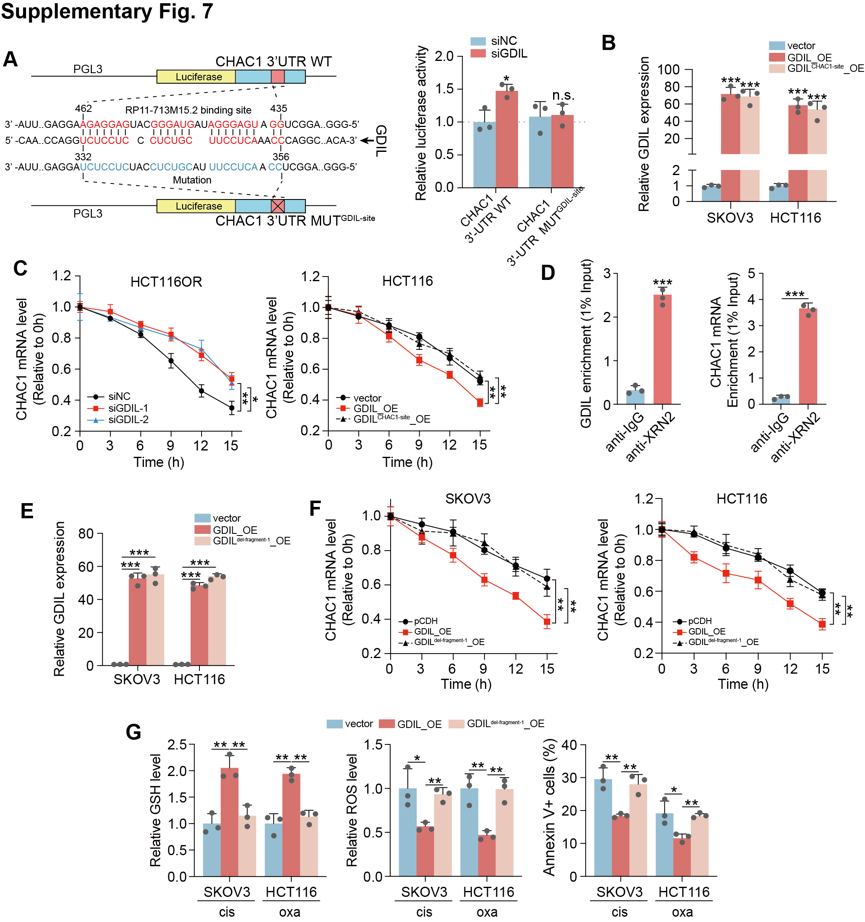


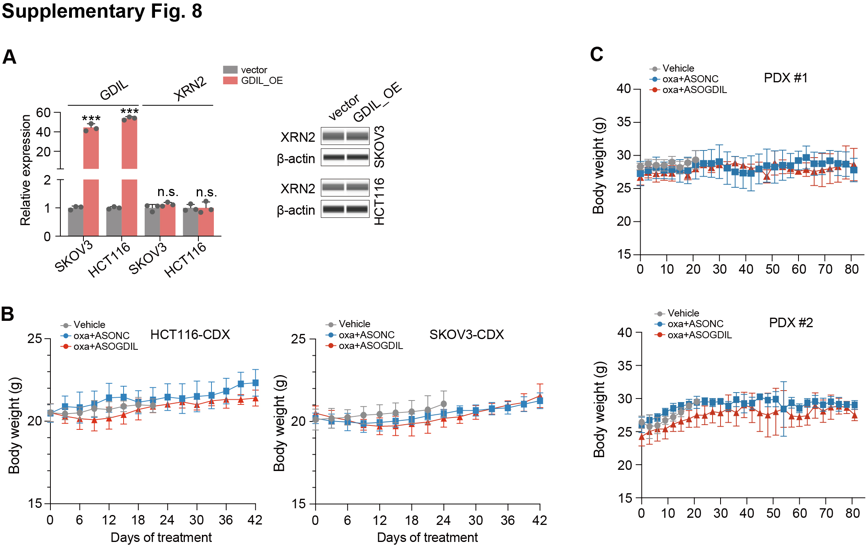

Supplement: Supplementary file 1 — Supplementary Figures [file 41419_2025_7374_MOESM1_ESM.docx]
