# Supplementary figures and images for: Long noncoding RNA GDIL acts as a scaffold for CHAC1 and XRN2 to promote platinum resistance of colorectal cancer through inhibition of glutathione degradation
Source: Cell Death Dis. 2025 Feb 1;16(1):62. doi: 10.1038/s41419-025-07374-w (PMC11787370; doi:10.1038/s41419-025-07374-w)

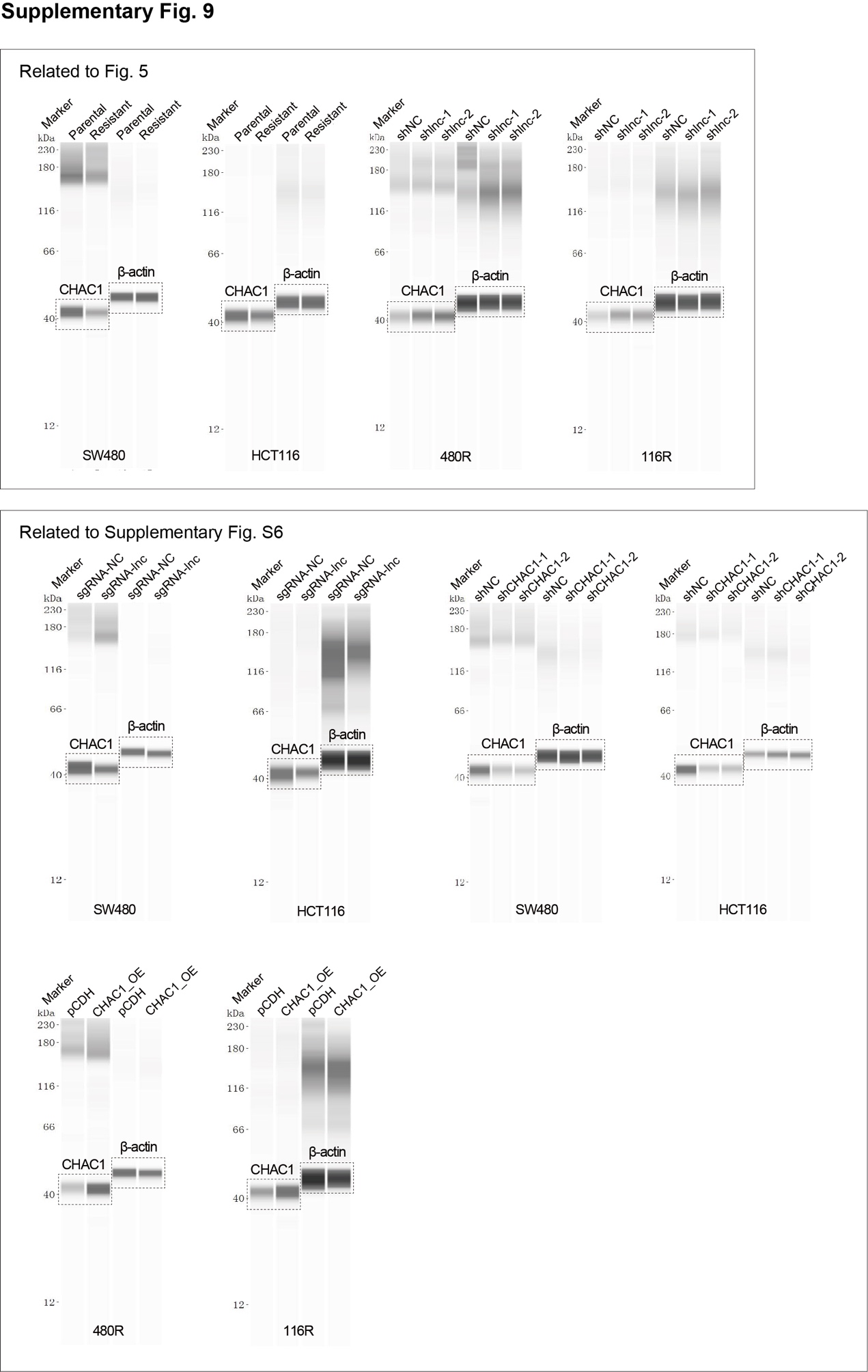

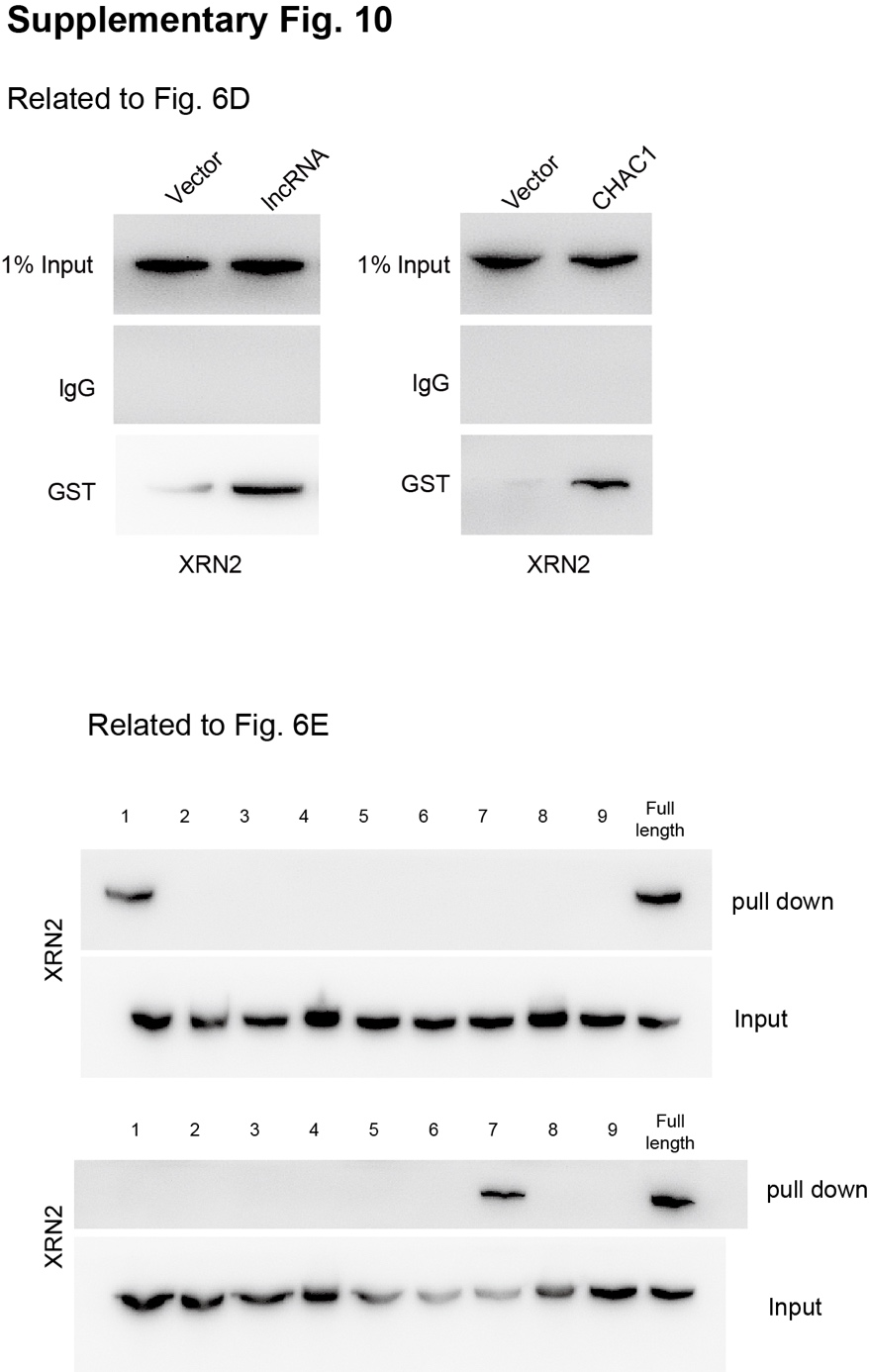

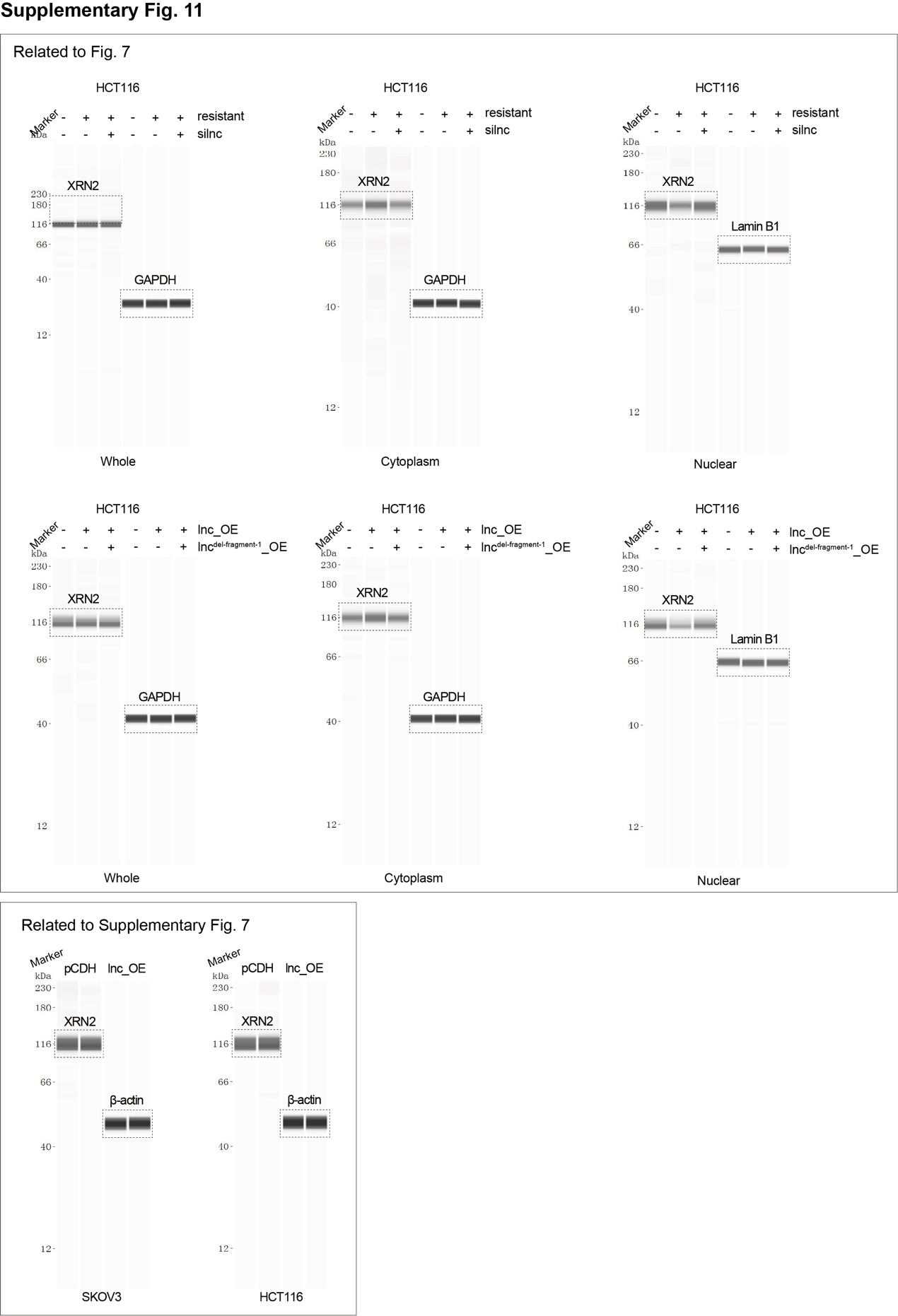

Supplement: Supplementary file 2 — Supplementary Figures-uncropped original blots [file 41419_2025_7374_MOESM2_ESM.docx]
