# Supplementary material for: Long noncoding RNA GDIL acts as a scaffold for CHAC1 and XRN2 to promote platinum resistance of colorectal cancer through inhibition of glutathione degradation
Source: Cell Death Dis. 2025 Feb 1;16(1):62. doi: 10.1038/s41419-025-07374-w (PMC11787370; doi:10.1038/s41419-025-07374-w)
Supplement: Supplementary file 3 — Supplementary Tables [file 41419_2025_7374_MOESM3_ESM.docx]

**Supplementary Table 1. Predicted peptides of GDIL.** Related to Supplementary Figure 1D.

| **Label** | **Frame** | **CDS**  **length** | **Peptide**  **length** | **Predicted peptides** |
| --- | --- | --- | --- | --- |
| ORF#1 | 3 | 153 | 50 | MLPGDLSFFLSSHIRSPPFKNSVGMGCISKVALQVSYLDFKLHFTRVTIL |
| ORF#2 | 2 | 174 | 57 | MKSQRSPSGLNLLSLNARNVFYRALDRCLCWPGVFNRSCLPQTPGTNVLP  ALTHSML |
| ORF#3 | 2 | 150 | 49 | MVHSRCFMLLSYVPFPLLSTLSPNTTQNKPTTVLLIPEVNPTQQGVERK |
| ORF#4 | 3 | 111 | 36 | MQEMFSIEPWTGVSAGLGFSTGHACLRPQGQMFFQL |
| ORF#5 | 1 | 84 | 27 | MGGFLYGNSGRKRDPQAAFGVALGKQR |
| ORF#6 | 2 | 81 | 26 | MGTQEEREIPRQHLVWHWENKDRSKA |
| ORF#7 | 3 | 150 | 49 | MWAFLDCSGKVNNHYNIVTLVKCNLKSK |
| ORF#8 | 3 | 273 | 90 | MKGKSAQEDRVRAPRRDARTRRRCSVGAHGVGELGRKGAAVGWREAGRGR  RSGSCPRGVVRSGDRGGEVWAGGWRVTAEAAAAGTGERPLG |

**Supplementary Table 2. Coding potential calculator based on sequence intrinsic features** (CPC, http://cpc2.cbi.pku.edu.cn/). Sequence of GDIL got Fickett score 0.27379 with a complete putative ORF 58 AA, a pI 10.125793457, which, in total, classify it as a noncoding sequence with coding probability 0.0309486.

| **LncRNA**  **/mRNA** | **Label** | **Peptide**  **length(aa)** | **Coding**  **probability** | **Fickett**  **score** | **Isoelectric point** |
| --- | --- | --- | --- | --- | --- |
| GDIL | Noncoding | 58 | 0.27379 | 0.27379 | 10.125793457 |
| MALAT1 | noncoding | 71 | 0.043828 | 0.27943 | 12.0846557617 |
| GAPDH | coding | 336 | 0.999999 | 0.4398 | 8.56610107422 |
| ACTB | coding | 376 | 1 | 0.44566 | 5.29046630859 |

**Supplementary Table 3. Recognizes coding and noncoding transcripts based on sequence intrinsic features** (CPAT, Coding Potential Assessing Tool, http://lilab.research.bcm.edu/).

| **LncRNA**  **/mRNA** | **ORF Size** | **Ficket**  **Score** | **Hexamer Score** | **Probability**  **Coding** | **Coding Label** |
| --- | --- | --- | --- | --- | --- |
| GDIL | 174 | 0.6805 | -0.1959 | 0.0043 | no |
| MALAT1 | 213 | 0.6529 | 0.0053 | 0.0142 | no |
| GAPDH | 1008 | 1.2926 | 0.5199 | 0.99996 | yes |
| ACTB | 1128 | 1.35 | 0.6975 | 0.999997 | yes |

**Supplementary Table 4. Clinical information of 90 CRC patients from Cohort 2 (fresh tissues)**

| 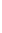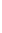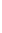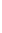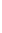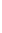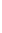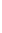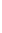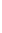   \| **Patients** \| \| --- \| | 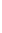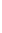   \| **Gender** \| \| --- \| | **Age** | 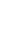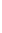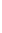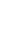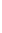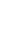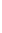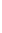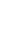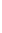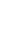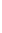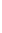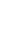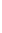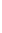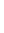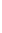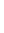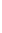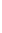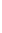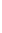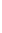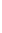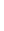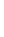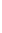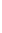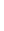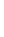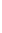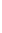   \| **Differentiation** \| \| --- \| | 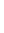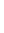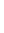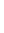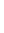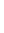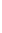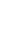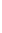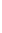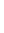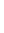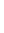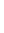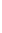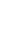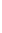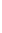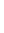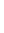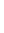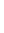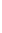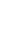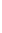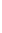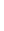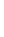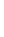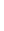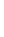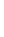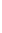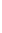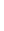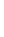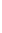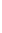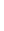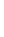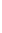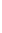   \| **Tumor**  **size** \| \| --- \| | **TNM stage** | **Therapy**  **Response** | **FolLow-up**  **time**  **(months)** | **GDIL**  **Expression (ΔCT=CT_GDIL_-CT_β-Actin_)** | **GDIL expression group** | **CHAC1 expression group** |
| --- | --- | --- | --- | --- | --- | --- | --- | --- | --- | --- | --- | --- | --- | --- |
| 1 | Male | 55 | Poor | 76 | II | SD | 25 | 14.15 | Low | Low |
| 2 | Male | 69 | Moderate | 105 | III | PD | 10 | 8.44 | High | Low |
| 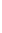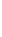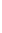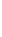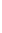   \| 3 \| \| --- \| | 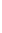   \| Male \| \| --- \| | 43 | Moderate | 30.625 | II | PD | 32 | 22.24 | Low | High |
| 4 | Female | 52 | Moderate | 1.125 | III | PD | 24 | 11.08 | High | Low |
| 5 | Female | 59 | Poor | 66.78 | III | PD | 6 | 9.92 | High | High |
| 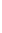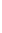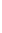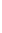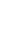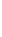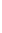   \| 6 \| \| --- \| | \| Male \| \| --- \| | 78 | Moderate | 207 | II | PD | 6 | 9.37 | High | Low |
| 7 | Male | 75 | Moderate | 59.4 | III | PD | 7 | 13.33 | Low | Low |
| 8 | Female | 39 | Moderate | \| 26.88 \| \| --- \| | III | PD | 12 | 16.72 | Low | High |
| 9 | Male | 42 | \| Moderate \| \| --- \| | 3.9 | III | PR | 60 | 9.08 | High | High |
| 10 | Male | 67 | Poor | 9 | II | PD | 15 | 11.16 | High | Low |
| 11 | Female | 55 | Poor | 59.85 | III | PR | 55 | 14.99 | Low | High |
| \| 12 \| \| --- \| | \| Male \| \| --- \| | 54 | Moderate | 21.824 | II | PD | 9 | 10.05 | High | Low |
| 13 | Male | 82 | Moderate | 5.88 | \| II \| \| --- \| | PD | \| 15 \| \| --- \| | 16.11 | Low | Low |
| \| 14 \| \| --- \| | \| Male \| \| --- \| | 80 | Poor | \| 84 \| \| --- \| | II | PD | 12 | 15.59 | Low | Low |
| 15 | Female | 58 | Moderate | 32.913 | III | PD | 18 | 12.35 | Low | High |
| \| 16 \| \| --- \| | \| Male \| \| --- \| | 66 | \| Moderate \| \| --- \| | 132.77 | II | PR | 31 | 12.57 | Low | High |
| 17 | Male | 62 | Moderate | \| 39.375 \| \| --- \| | III | PD | 8 | 10.93 | High | Low |
| 18 | Male | 53 | Poor | 142.625 | III | SD | 59 | 14.41 | Low | High |
| 19 | Female | 77 | Poor | 11.2 | II | PR | 29 | 6.97 | High | High |
| 20 | Female | 54 | Poor | 12.6 | \| III \| \| --- \| | SD | \| 28 \| \| --- \| | 9.32 | High | High |
| \| 21 \| \| --- \| | \| Female \| \| --- \| | 80 | Moderate | \| 49.5 \| \| --- \| | III | PD | 11 | 8.88 | High | Low |
| 22 | Male | 66 | \| Moderate \| \| --- \| | 9.92 | \| III \| \| --- \| | PD | \| 8 \| \| --- \| | 11.14 | High | Low |
| 23 | Male | 63 | Poor | 11.52 | II | PR | 33 | 14.36 | Low | Low |
| 24 | Male | 68 | Moderate | 71.4 | III | PR | 45 | 10.12 | High | High |
| \| 25 \| \| --- \| | \| Male \| \| --- \| | 59 | \| Poor \| \| --- \| | 10.648 | III | PD | 9 | 16.23 | Low | Low |
| 26 | Male | 44 | Moderate | 4.95 | III | CR | 44 | 15.53 | Low | High |
| 27 | Female | 52 | Moderate | 24 | II | SD | 60 | 15.81 | Low | High |
| 28 | Male | 47 | Moderate | 10.5 | II | PD | 23 | 12.43 | Low | Low |
| 29 | Male | 76 | Poor | 45.056 | III | PD | 16 | 12.05 | Low | Low |
| 30 | Male | 65 | Poor | \| 19.8 \| \| --- \| | \| III \| \| --- \| | PD | \| 8 \| \| --- \| | 7.11 | High | Low |
| \| 31 \| \| --- \| | \| Male \| \| --- \| | 83 | \| Moderate \| \| --- \| | 44.16 | III | SD | 23 | 11.19 | High | Low |
| 32 | Male | 77 | Poor | 40.95 | II | PD | 10 | 10.25 | High | High |
| 33 | Male | 71 | Moderate | 14.56 | \| II \| \| --- \| | PD | \| 18 \| \| --- \| | 16.21 | Low | High |
| \| 34 \| \| --- \| | \| Female \| \| --- \| | 59 | \| Moderate \| \| --- \| | 20.988 | II | PR | 37 | 13.36 | Low | High |
| 35 | Female | 67 | Poor | 23.52 | \| III \| \| --- \| | SD | \| 35 \| \| --- \| | 8.19 | High | Low |
| 36 | Male | 80 | \| Poor \| \| --- \| | 30 | II | SD | 20 | 10.88 | High | Low |
| 37 | Male | 65 | Moderate | 7.5 | II | CR | 60 | 15.02 | Low | High |
| \| 38 \| \| --- \| | \| Male \| \| --- \| | 58 | Moderate | 69.56 | \| II \| \| --- \| | PD | \| 12 \| \| --- \| | 9.28 | High | Low |
| 39 | Female | 54 | Moderate | 21.624 | III | PD | 14 | 13.34 | Low | High |
| 40 | Female | 48 | Moderate | 43.12 | III | PD | 15 | 8.43 | High | Low |
| \| 41 \| \| --- \| | \| Female \| \| --- \| | 63 | Moderate | 23.52 | III | PR | 51 | 14.42 | Low | Low |
| 42 | Male | 65 | Poor | 7.2 | \| III \| \| --- \| | PD | \| 14 \| \| --- \| | 8.77 | High | Low |
| 43 | Male | 78 | Poor | 72.8 | III | PR | 32 | 5.52 | High | High |
| \| 44 \| \| --- \| | \| Male \| \| --- \| | 43 | Poor | 40.7 | II | PR | 56 | 15.17 | Low | High |
| 45 | Male | 55 | Moderate | 18.2 | II | SD | 47 | 12.33 | Low | High |
| 46 | Male | 52 | \| Moderate \| \| --- \| | 15.708 | II | PD | 8 | 11.94 | High | High |
| 47 | Male | 68 | Moderate | 46.2 | \| II \| \| --- \| | PR | \| 36 \| \| --- \| | 13.26 | Low | High |
| 48 | Female | 67 | Moderate | 2.43 | III | PR | 47 | 14.90 | Low | High |
| \| 49 \| \| --- \| | \| Male \| \| --- \| | 73 | Moderate | 16.456 | III | PD | 17 | 6.23 | High | Low |
| 50 | Male | 75 | Moderate | 4.83 | II | PD | 26 | 8.89 | High | Low |
| 51 | Male | 47 | Poor | 18.117 | III | PD | 14 | 15.02 | Low | Low |
| \| 52 \| \| --- \| | \| Male \| \| --- \| | 59 | \| Poor \| \| --- \| | 13.5 | II | SD | 55 | 16.63 | Low | High |
| 53 | Female | 62 | Moderate | 13.3 | III | PD | 18 | 10.51 | High | Low |
| 54 | Male | 71 | Moderate | 168 | \| III \| \| --- \| | PD | \| 6 \| \| --- \| | 8.37 | High | Low |
| 55 | Male | 59 | Poor | 2.3 | III | PD | 7 | 13.80 | Low | Low |
| 56 | Male | 66 | Poor | 19.74 | II | PR | 42 | 14.43 | Low | High |
| 57 | Male | 64 | Poor | \| 97.76 \| \| --- \| | III | PR | 38 | 11.07 | High | High |
| 58 | Female | 72 | Moderate | 42 | III | PR | 29 | 13.32 | Low | High |
| 59 | Male | 63 | Moderate | 12.65 | II | SD | 39 | 15.55 | Low | High |
| \| 60 \| \| --- \| | \| Male \| \| --- \| | 65 | Moderate | 13.125 | \| II \| \| --- \| | SD | \| 45 \| \| --- \| | 16.78 | Low | High |
| 61 | Female | 62 | Poor | 16 | II | CR | 43 | 11.23 | High | High |
| 62 | Female | 55 | Moderate | \| 8.4375 \| \| --- \| | III | SD | 55 | 6.21 | High | High |
| 63 | Male | 73 | Poor | 116.56 | II | SD | 33 | 17.77 | Low | Low |
| \| 64 \| \| --- \| | \| Male \| \| --- \| | 64 | Moderate | 37.44 | \| II \| \| --- \| | PR | \| 49 \| \| --- \| | 15.65 | Low | High |
| 65 | Male | 54 | Poor | 9.6 | III | PD | 12 | 7.03 | High | Low |
| 66 | Male | 49 | Poor | 7.8 | \| III \| \| --- \| | PD | \| 22 \| \| --- \| | 6.32 | High | Low |
| 67 | Male | 45 | Moderate | 13.5 | II | SD | 58 | 12.66 | Low | High |
| 68 | Male | 62 | \| Moderate \| \| --- \| | 30.21 | \| III \| \| --- \| | PR | \| 42 \| \| --- \| | 10.38 | High | High |
| \| 69 \| \| --- \| | \| Male \| \| --- \| | 54 | Poor | 11.088 | III | PD | 20 | 13.48 | Low | High |
| 70 | Male | 58 | Poor | 9.408 | II | PD | 14 | 8.63 | High | Low |
| 71 | Male | 51 | Poor | 1.08 | \| II \| \| --- \| | CR | \| 38 \| \| --- \| | 14.17 | Low | High |
| 72 | Male | 68 | Moderate | 20.25 | III | PD | 21 | 16.89 | Low | Low |
| 73 | Male | 76 | Moderate | 4 | II | PD | 10 | 8.42 | High | Low |
| 74 | Male | 77 | Moderate | 36.54 | \| III \| \| --- \| | SD | \| 31 \| \| --- \| | 10.28 | High | Low |
| \| 75 \| \| --- \| | \| Male \| \| --- \| | 56 | Moderate | 9.92 | II | PR | 58 | 14.94 | Low | High |
| 76 | Female | 59 | Moderate | 6.6 | II | SD | 45 | 11.46 | High | High |
| \| 77 \| \| --- \| | \| Female \| \| --- \| | 60 | Poor | 13.932 | \| II \| \| --- \| | SD | \| 39 \| \| --- \| | 12.78 | Low | High |
| 78 | Male | 63 | Poor | 26.4 | III | SD | 36 | 9.65 | High | Low |
| 79 | \| Male \| \| --- \| | 78 | \| Moderate \| \| --- \| | 36.96 | \| III \| \| --- \| | PD | 10 | 6.02 | High | High |
| 80 | Male | 73 | Moderate | 112.5 | II | SD | 32 | 10.24 | High | Low |
| 81 | \| Male \| \| --- \| | 52 | Moderate | 5.166 | \| II \| \| --- \| | PD | 35 | 9.99 | High | Low |
| 82 | Male | 32 | Poor | 27.2 | II | SD | 51 | 15.78 | Low | Low |
| \| 83 \| \| --- \| | \| Male \| \| --- \| | 45 | \| Moderate \| \| --- \| | 8.96 | II | SD | 52 | 13.12 | Low | High |
| 84 | Female | 48 | Poor | 0.6 | II | PR | 54 | 9.08 | High | Low |
| 85 | Male | 87 | Moderate | 28.352 | \| III \| \| --- \| | PD | 11 | 9.55 | High | Low |
| 86 | Male | 83 | Moderate | 35.1 | III | PD | 16 | 16.09 | Low | High |
| \| 87 \| \| --- \| | Male | 75 | Moderate | 67.2 | II | PD | \| 29 \| \| --- \| | 15.23 | Low | High |
| 88 | \| Male \| \| --- \| | 64 | Poor | 6.25 | \| II \| \| --- \| | PD | 32 | 10.44 | High | Low |
| 89 | Female | 69 | Poor | \| 2.4 \| \| --- \| | II | PR | 38 | 14.43 | Low | High |
| \| 90 \| \| --- \| | \| Male \| \| --- \| | 71 | \| Moderate \| \| --- \| | 41.664 | \| III \| \| --- \| | PD | \| 17 \| \| --- \| | 10.77 | High | Low |

*Treatment response was assessed with morphologic imaging (CT) and classified according to the response evaluation criteria in solid tumors guideline into complete response (CR), partial response (PR), stable disease (SD), and progressive disease (PD).

**Supplementary Table 5. Proteins specifically bind with sense GDIL (compared with anti-sense GDIL) analyzed by RNA pull down and Mass spectrometry.**

| Accession | Protein name | SKOV3 | | HCT116 | |
| --- | --- | --- | --- | --- | --- |
|  |  | #Peptides | #Unique | #Peptides | #Unique |
| Q8WVV9 | Heterogeneous nuclear ribonucleoprotein L-like (HNRNPLL) | 8 | 7 | 1 | 1 |
| **Q9H0D6** | **5'-3' exoribonuclease 2 (XRN2)** | **7** | **7** | **1** | **1** |
| Q96P70 | Importin-9 (IPO9) | 6 | 6 | 1 | 1 |
| Q6PD62 | RNA polymerase-associated protein CTR9 homolog (CTR9) | 6 | 6 | 1 | 1 |
| Q15642 | Cdc42-interacting protein 4 (TRIP10) | 5 | 5 | 1 | 1 |
| P61421 | V-type proton ATPase subunit d 1 (ATP6V0D1) | 5 | 5 | 1 | 1 |
| P19784 | Casein kinase II subunit alpha' (CSNK2A2) | 6 | 5 | 5 | 4 |
| Q53GS9 | U4/U6.U5 tri-snRNP-associated protein 2 (USP39) | 5 | 5 | 3 | 3 |
| Q92544 | Transmembrane 9 superfamily member 4 (TM9SF4) | 4 | 4 | 1 | 1 |
| Q9UI26 | Importin-11 (IPO11) | 4 | 4 | 1 | 1 |
| Q9Y2Z4 | Tyrosine--tRNA ligase mitochondrial (YARS2) | 4 | 4 | 1 | 1 |
| Q9Y305 | Acyl-coenzyme A thioesterase 9 mitochondrial (ACOT9) | 4 | 4 | 5 | 5 |
| P27361 | Mitogen-activated protein kinase 3 (MAPK3) | 4 | 4 | 1 | 1 |
| O15067 | Phosphoribosylformylglycinamidine synthase (PFAS) | 4 | 4 | 1 | 1 |
| P29992 | Guanine nucleotide-binding protein subunit alpha-11 (GNA11) | 4 | 4 | 2 | 2 |
| Q14694 | Ubiquitin carboxyl-terminal hydrolase 10 (USP10) | 4 | 4 | 1 | 1 |
| Q14CX7 | N-alpha-acetyltransferase 25 NatB auxiliary subunit (NAA25) | 4 | 4 | 2 | 2 |
| Q9Y2S7 | Polymerase delta-interacting protein 2 (POLDIP2) | 4 | 4 | 1 | 1 |
| P63172 | Dynein light chain Tctex-type 1 (DYNLT1) | 3 | 3 | 2 | 2 |
| Q14376 | UDP-glucose 4-epimerase (GALE) | 3 | 3 | 2 | 2 |
| Q8NF37 | Lysophosphatidylcholine acyltransferase 1 (LPCAT1) | 3 | 3 | 3 | 3 |
| P17174 | Aspartate aminotransferase cytoplasmic (GOT1) | 3 | 3 | 7 | 7 |
| Q15006 | ER membrane protein complex subunit 2 (EMC2) | 3 | 3 | 1 | 1 |
| P49841 | Glycogen synthase kinase-3 beta (GSK3B) | 3 | 3 | 1 | 1 |
| Q92896 | Golgi apparatus protein 1 (GLG1) | 3 | 3 | 4 | 4 |
| Q14978 | Nucleolar and coiled-body phosphoprotein 1 (NOLC1) | 3 | 3 | 1 | 1 |
| Q9H9T3 | Elongator complex protein 3 (ELP3) | 3 | 3 | 1 | 1 |
| Q96JB2 | Conserved oligomeric Golgi complex subunit 3 (COG3) | 3 | 3 | 1 | 1 |
| Q8WWM7 | Ataxin-2-like protein (ATXN2L) | 3 | 3 | 1 | 1 |
| Q9Y295 | Developmentally-regulated GTP-binding protein 1 (DRG1) | 3 | 3 | 3 | 3 |
| Q9H2M9 | Rab3 GTPase-activating protein non-catalytic subunit (RAB3GAP2) | 3 | 3 | 1 | 1 |
| O95747 | Serine/threonine-protein kinase OSR1 (OXSR1) | 3 | 3 | 1 | 1 |
| Q9BZX2 | Uridine-cytidine kinase 2 (UCK2) | 3 | 3 | 1 | 1 |
| Q06203 | Amidophosphoribosyltransferase (PPAT) | 3 | 3 | 4 | 4 |
| O75152 | Zinc finger CCCH domain-containing protein 11A (ZC3H11A) | 3 | 3 | 1 | 1 |
| Q9Y6M1 | Insulin-like growth factor 2 mRNA-binding protein 2 (IGF2BP2) | 4 | 2 | 4 | 4 |
| P62837 | Ubiquitin-conjugating enzyme E2 D2 (UBE2D2) | 2 | 2 | 2 | 2 |
| Q9UPN7 | Serine/threonine-protein phosphatase 6 regulatory subunit 1 (PPP6R1) | 2 | 2 | 1 | 1 |
| Q96GA7 | Serine dehydratase-like (SDSL) | 2 | 2 | 1 | 1 |
| Q9HB71 | Calcyclin-binding protein (CACYBP) | 2 | 2 | 5 | 5 |
| O94874 | E3 UFM1-protein ligase 1 (UFL1) | 2 | 2 | 1 | 1 |
| Q9NX24 | H/ACA ribonucleoprotein complex subunit 2 (NHP2) | 2 | 2 | 1 | 1 |
| Q99615 | DnaJ homolog subfamily C member 7 (DNAJC7) | 2 | 2 | 2 | 2 |
| Q5RI15 | Cytochrome c oxidase assembly protein COX20 mitochondrial (COX20) | 2 | 2 | 1 | 1 |
| Q5VWZ2 | Lysophospholipase-like protein 1 (LYPLAL1) | 2 | 2 | 1 | 1 |
| Q9NUQ9 | Protein FAM49B (FAM49B) | 2 | 2 | 1 | 1 |
| Q8IXM3 | 39S ribosomal protein L41 mitochondrial (MRPL41) | 2 | 2 | 1 | 1 |
| Q05519 | Serine/arginine-rich splicing factor 11 (SRSF11) | 2 | 2 | 1 | 1 |
| Q9Y5L4 | Mitochondrial import inner membrane translocase subunit Tim13 (TIMM13) | 2 | 2 | 2 | 2 |
| Q9UPT8 | Zinc finger CCCH domain-containing protein 4 (ZC3H4) | 2 | 2 | 2 | 2 |
| P11233 | Ras-related protein Ral-A (RALA) | 2 | 2 | 3 | 3 |
| Q969X6 | U3 small nucleolar RNA-associated protein 4 homolog (UTP4) | 2 | 2 | 2 | 2 |
| P42345 | Serine/threonine-protein kinase mTOR (MTOR) | 2 | 2 | 1 | 1 |
| Q9NRY5 | Protein FAM114A2 (FAM114A2) | 2 | 2 | 2 | 2 |
| Q92665 | 28S ribosomal protein S31 mitochondrial (MRPS31) | 2 | 2 | 1 | 1 |
| P78346 | Ribonuclease P protein subunit p30 (RPP30) | 2 | 2 | 1 | 1 |
| P82673 | 28S ribosomal protein S35 mitochondrial (MRPS35) | 2 | 2 | 1 | 1 |
| P62306 | Small nuclear ribonucleoprotein F (SNRPF) | 2 | 2 | 3 | 3 |
| P37268 | Squalene synthase (FDFT1) | 2 | 2 | 2 | 2 |
| Q99797 | Mitochondrial intermediate peptidase (MIPEP) | 2 | 2 | 1 | 1 |
| O14776 | Transcription elongation regulator 1 (TCERG1) | 2 | 2 | 1 | 1 |
| Q9ULC5 | Long-chain-fatty-acid--CoA ligase 5 (ACSL5) | 2 | 2 | 16 | 16 |
| P36404 | ADP-ribosylation factor-like protein 2 (ARL2) | 2 | 2 | 1 | 1 |
| P10316 | HLA class I histocompatibility antigen A-69 alpha chain (HLA-A) | 8 | 1 | 7 | 1 |
| P61020 | Ras-related protein Rab-5B (RAB5B) | 4 | 1 | 3 | 1 |
| P09972 | Fructose-bisphosphate aldolase C (ALDOC) | 2 | 1 | 1 | 1 |
| Q01082 | Spectrin beta chain non-erythrocytic 1 (SPTBN1) | 1 | 1 | 3 | 3 |
| Q9UHL4 | Dipeptidyl peptidase 2 (DPP7) | 1 | 1 | 2 | 2 |
| Q03252 | Lamin-B2 (LMNB2) | 2 | 1 | 6 | 5 |
| Q96QD8 | Sodium-coupled neutral amino acid transporter 2 (SLC38A2) | 1 | 1 | 1 | 1 |
| Q9P003 | Protein cornichon homolog 4 (CNIH4) | 1 | 1 | 1 | 1 |
| Q8N138 | ORM1-like protein 3 (ORMDL3) | 1 | 1 | 1 | 1 |
| P14854 | Cytochrome c oxidase subunit 6B1 (COX6B1) | 1 | 1 | 2 | 2 |
| Q96T88 | E3 ubiquitin-protein ligase UHRF1 (UHRF1) | 1 | 1 | 2 | 2 |
| P29372 | DNA-3-methyladenine glycosylase (MPG) | 1 | 1 | 1 | 1 |
| Q9NPE3 | H/ACA ribonucleoprotein complex subunit 3 (NOP10) | 1 | 1 | 1 | 1 |
| Q14677 | Clathrin interactor 1 (CLINT1) | 1 | 1 | 1 | 1 |
| O75947 | ATP synthase subunit d mitochondrial (ATP5PD) | 1 | 1 | 1 | 1 |
| P41214 | Eukaryotic translation initiation factor 2D (EIF2D) | 1 | 1 | 1 | 1 |
| Q9Y639 | Neuroplastin (NPTN) | 1 | 1 | 1 | 1 |
| Q13616 | Cullin-1 (CUL1) | 1 | 1 | 1 | 1 |
| Q9UHV9 | Prefoldin subunit 2 (PFDN2) | 1 | 1 | 2 | 2 |
| P63279 | SUMO-conjugating enzyme UBC9 (UBE2I) | 1 | 1 | 3 | 3 |
| Q96LD4 | E3 ubiquitin-protein ligase TRIM47 (TRIM47) | 1 | 1 | 1 | 1 |
| Q9H8Y8 | Golgi reassembly-stacking protein 2 (GORASP2) | 1 | 1 | 1 | 1 |
| Q92572 | AP-3 complex subunit sigma-1 (AP3S1) | 1 | 1 | 2 | 2 |
| Q4G176 | Acyl-CoA synthetase family member 3 mitochondrial (ACSF3) | 1 | 1 | 1 | 1 |
| O43488 | Aflatoxin B1 aldehyde reductase member 2 (AKR7A2) | 1 | 1 | 2 | 2 |
| P07947 | Tyrosine-protein kinase Yes (YES1) | 2 | 1 | 3 | 2 |
| Q14728 | Major facilitator superfamily domain-containing protein 10 (MFSD10) | 1 | 1 | 1 | 1 |
| P13987 | CD59 glycoprotein (CD59) | 1 | 1 | 2 | 2 |
| Q15042 | Rab3 GTPase-activating protein catalytic subunit (RAB3GAP1) | 1 | 1 | 1 | 1 |
| O14497 | AT-rich interactive domain-containing protein 1A (ARID1A) | 1 | 1 | 1 | 1 |
| Q9HD33 | 39S ribosomal protein L47 mitochondrial (MRPL47) | 1 | 1 | 1 | 1 |
| Q96JH7 | Deubiquitinating protein VCIP135 (VCPIP1) | 1 | 1 | 1 | 1 |
| O96019 | Actin-like protein 6A (ACTL6A) | 1 | 1 | 1 | 1 |
| O95777 | U6 snRNA-associated Sm-like protein LSm8 (LSM8) | 1 | 1 | 1 | 1 |
| Q9NYT0 | Pleckstrin-2 (PLEK2) | 1 | 1 | 1 | 1 |
| Q8N122 | Regulatory-associated protein of mTOR (RPTOR) | 1 | 1 | 1 | 1 |
| P11802 | Cyclin-dependent kinase 4 (CDK4) | 2 | 1 | 1 | 1 |
| P41743 | Protein kinase C iota type (PRKCI) | 1 | 1 | 1 | 1 |
| Q59GN2 | Putative 60S ribosomal protein L39-like 5 (RPL39P5 PE=5 SV=2 | 1 | 1 | 1 | 1 |
| P62891 | 60S ribosomal protein L39 (RPL39) | 1 | 1 | 1 | 1 |
| Q9NQ88 | Fructose-2 6-bisphosphatase TIGAR (TIGAR) | 1 | 1 | 1 | 1 |
| P63208 | S-phase kinase-associated protein 1 (SKP1) | 1 | 1 | 2 | 2 |
| O00625 | Pirin (PIR) | 1 | 1 | 1 | 1 |
| Q9UBQ0 | Vacuolar protein sorting-associated protein 29 (VPS29) | 1 | 1 | 1 | 1 |
| Q9H0U6 | 39S ribosomal protein L18 mitochondrial (MRPL18) | 1 | 1 | 1 | 1 |
| Q9BZH6 | WD repeat-containing protein 11 (WDR11) | 1 | 1 | 1 | 1 |
| Q9NP92 | 39S ribosomal protein S30 mitochondrial (MRPS30) | 1 | 1 | 1 | 1 |
| Q9BPW8 | Protein NipSnap homolog 1 (NIPSNAP1) | 1 | 1 | 4 | 4 |
| Q969M3 | Protein YIPF5 (YIPF5) | 1 | 1 | 1 | 1 |
| P55210 | Caspase-7 (CASP7) | 1 | 1 | 1 | 1 |
| Q8N7H5 | RNA polymerase II-associated factor 1 homolog (PAF1) | 1 | 1 | 2 | 2 |
| Q92797 | Symplekin (SYMPK) | 1 | 1 | 2 | 2 |
| P22307 | Non-specific lipid-transfer protein (SCP2) | 1 | 1 | 1 | 1 |
| Q96RN5 | Mediator of RNA polymerase II transcription subunit 15 (MED15) | 1 | 1 | 1 | 1 |
| Q96HE7 | ERO1-like protein alpha (ERO1A) | 1 | 1 | 8 | 8 |
| Q9NQT8 | Kinesin-like protein KIF13B (KIF13B) | 1 | 1 | 1 | 1 |
| Q6NUM9 | All-trans-retinol 13 14-reductase (RETSAT) | 1 | 1 | 1 | 1 |
| O43688 | Phospholipid phosphatase 2 (PLPP2) | 1 | 1 | 2 | 2 |
| Q9UBV2 | Protein sel-1 homolog 1 (SEL1L) | 1 | 1 | 1 | 1 |
| P05161 | Ubiquitin-like protein ISG15 (ISG15) | 1 | 1 | 4 | 4 |
| P49406 | 39S ribosomal protein L19 mitochondrial (MRPL19) | 1 | 1 | 2 | 2 |
| Q04760 | Lactoylglutathione lyase (GLO1) | 1 | 1 | 4 | 4 |
| P35251 | Replication factor C subunit 1 (RFC1) | 1 | 1 | 1 | 1 |
| P46926 | Glucosamine-6-phosphate isomerase 1 (GNPDA1) | 1 | 1 | 2 | 2 |
| Q7L5D6 | Golgi to ER traffic protein 4 homolog (GET4) | 1 | 1 | 1 | 1 |
| P61326 | Protein mago nashi homolog (MAGOH) | 1 | 1 | 1 | 1 |
| Q96A72 | Protein mago nashi homolog 2 (MAGOHB) | 1 | 1 | 1 | 1 |
| O75907 | Diacylglycerol O-acyltransferase 1 (DGAT1) | 1 | 1 | 1 | 1 |
| Q96E29 | Transcription termination factor 3 mitochondrial (MTERF3) | 1 | 1 | 1 | 1 |
| Q96JM3 | Chromosome alignment-maintaining phosphoprotein 1 (CHAMP1) | 1 | 1 | 1 | 1 |
| P02795 | Metallothionein-2 (MT2A) | 1 | 1 | 3 | 3 |
| P09001 | 39S ribosomal protein L3 mitochondrial (MRPL3) | 1 | 1 | 1 | 1 |
| P30046 | D-dopachrome decarboxylase (DDT) | 1 | 1 | 3 | 3 |
| Q9NX20 | 39S ribosomal protein L16 mitochondrial (MRPL16) | 1 | 1 | 1 | 1 |
| Q92990 | Glomulin (GLMN) | 1 | 1 | 1 | 1 |
| P33527 | Multidrug resistance-associated protein 1 (ABCC1) | 1 | 1 | 1 | 1 |
| P11047 | Laminin subunit gamma-1 (LAMC1) | 1 | 1 | 1 | 1 |
| Q99471 | Prefoldin subunit 5 (PFDN5) | 1 | 1 | 2 | 2 |
| Q9HCG8 | Pre-mRNA-splicing factor CWC22 homolog (CWC22) | 1 | 1 | 1 | 1 |
| P18858 | DNA ligase 1 (LIG1) | 1 | 1 | 2 | 2 |
| Q9BUN8 | Derlin-1 (DERL1) | 1 | 1 | 1 | 1 |
| Q9Y5P6 | Mannose-1-phosphate guanyltransferase beta (GMPPB) | 1 | 1 | 2 | 2 |
| P30405 | Peptidyl-prolyl cis-trans isomerase F mitochondrial (PPIF) | 1 | 1 | 1 | 1 |
| P13807 | Glycogen [starch] synthase muscle (GYS1) | 1 | 1 | 1 | 1 |
| Q86U90 | YrdC domain-containing protein mitochondrial (YRDC) | 1 | 1 | 1 | 1 |
| O43913 | Origin recognition complex subunit 5 (ORC5) | 1 | 1 | 1 | 1 |
| Q9UKV8 | Protein argonaute-2 (AGO2) | 1 | 1 | 1 | 1 |
| Q96HJ9 | Protein FMC1 homolog (FMC1) | 1 | 1 | 1 | 1 |
| P51532 | Transcription activator BRG1 (SMARCA4) | 1 | 1 | 1 | 1 |
| P51531 | Probable global transcription activator SNF2L2 (SMARCA2) | 1 | 1 | 1 | 1 |
| P06396 | Gelsolin (GSN) | 1 | 1 | 2 | 2 |
| P49916 | DNA ligase 3 (LIG3) | 1 | 1 | 2 | 2 |
| P23193 | Transcription elongation factor A protein 1 (TCEA1) | 1 | 1 | 1 | 1 |
| P00568 | Adenylate kinase isoenzyme 1 (AK1) | 1 | 1 | 1 | 1 |
| Q9Y399 | 28S ribosomal protein S2 mitochondrial (MRPS2) | 1 | 1 | 1 | 1 |
| P10606 | Cytochrome c oxidase subunit 5B mitochondrial (COX5B) | 1 | 1 | 3 | 3 |
| Q8NFH5 | Nucleoporin NUP35 (NUP35) | 1 | 1 | 1 | 1 |
| Q92625 | Ankyrin repeat and SAM domain-containing protein 1A (ANKS1A) | 1 | 1 | 1 | 1 |

**Supplementary Table 6. IC_50_ values of cell lines in this study.**

| **Cell lines** | **Initial IC_50_** | **Final IC_50_** | **Drug resistance index (the ratio of IC_50_ values between resistant and parental cells)** |
| --- | --- | --- | --- |
| **SW480 (oxaliplatin)** | 0.845 µM | 11.850 µM | 14.024 |
| **HCT116**  **(oxaliplatin)** | 4.013 µM | 25.293 µM | 6.303 |
| **C4**  **(oxaliplatin)** | 1.783 µM | 8.559 µM | 4.800 |
| **R21**  **(oxaliplatin)** | 1.412 µM | 9.023 µM | 6.390 |
| **SKOV3**  **(cisplatin)** | 2.303 µM | 20.215 µM | 8.778 |

**Supplementary Table 7. Primers used in this study.**

| **Primers used for quantitative RT-PCR** | | |
| --- | --- | --- |
| **Name** | **Forward** | **Reverse** |
| GDIL | ACCCAGCCCGTAGAATTGTC | GTAAGAACTCCGCAGAGGCA |
| ROBO1 | CCTGTTTCTGGCCCAGCTTA | GCCTGTATAGCCCAGCGAAT |
| GRM8 | CCATGGTGGACATCGTGACA | GGTCTTGGTTCACGTGGGAT |
| CXCL8 | GAGAGTGATTGAGAGTGGACCAC | CACAACCCTCTGCACCCAGTTT |
| TXNIP | CAGCAGTGCAAACAGACTTCGG | CTGAGGAAGCTCAAAGCCGAAC |
| LUCAT1 | GGATGAGACTTAGCGTGCCT | CCTCGGGTTGCCTCTGTTTA |
| GPX2 | TGGCTTCCCTTGCAACCAAT | GTGAAGGTGGGCTGGTATCC |
| GAPDH | TCACCACCA TGGAGAAGGC | GCTAAGCAGTTGGTGGTGCA |
| U6 | CGCTTCGGCAGCACATATA | TTCACGAA TTTGCGTGTCAT |
| β-actin | AGTTGCGTTACACCCTTTCTTG | GCTGTCACCTTCACCGTTCC |
| CHAC1 | GTGGTGACGCTCCTTGAAGATC | GAAGGTGACCTCCTTGGTATCG |
| **Primers used for RACE-PCR** | | |
| **Name** | **Sequence** |  |
| lncRNA-5′ RACE-GSP | GAACTCAGGGTGGTTCAGGTGTGACATGGG | |
| lncRNA-5′ RACE-NGSP | CCAGGCAGCATTTGGTGTGGCACTGGG | |
| lncRNA-3′ RACE-GSP | GAGCCGACTTCCCTTTCATACCCAGCCCG | |
| lncRNA-3′ RACE-NGSP | GGCTTCGCTGCCTCTGCGGAGTTCTTAC | |

**Supplementary Table 8. Antibodies used in this study**

| **Antibodies for immunoblotting** | | | | |
| --- | --- | --- | --- | --- |
| **Antibody** | **Company** | **Catalog #** | **Species** | **Dilution/**  **Concentration** |
| Lamin B1 | Cell signaling | 13435 | Rabbit | 1:1000 |
| GAPDH | Cell signaling | 2118 | Rabbit | 1:1000 |
| β-actin | Proteintech | HRP-60008 | Mouse | 1:10000 |
| CHAC1 | Proteintech | 15207-1-AP | Rabbit | 1:1000 |
| XRN2 | Cell signaling | 13760 | Rabbit | 1:1000 |
| **Antibodies for IHC** | | | | |
| CHAC1 | Abcam | ab279365 | Mouse | 1:150 |
| Ki67 | Abcam | ab15580 | Rabbit | 1µg/ml |
| Caspase-3 | Cell signaling | 9662 | Rabbit | 1:1000 |
| **Antibodies for RIP** | | | | |
| XRN2 | Cell signaling | 13760 | Rabbit | 1:100 |
