# Supplementary material for: Long noncoding RNA GDIL acts as a scaffold for CHAC1 and XRN2 to promote platinum resistance of colorectal cancer through inhibition of glutathione degradation
Source: Cell Death Dis. 2025 Feb 1;16(1):62. doi: 10.1038/s41419-025-07374-w (PMC11787370; doi:10.1038/s41419-025-07374-w)
Supplement: Supplementary file 4 — Supplementary Materials and Methods [file 41419_2025_7374_MOESM4_ESM.docx]

**Supplementary** **Materials and Methods**

**Data collection**

The clinical data and correlated mRNA expression of COAD, READ and normal cases were obtained from The Cancer Genome Atlas (TCGA).

**Isolation of primary CRC cell lines**

All reagents used for cell culture were purchased from Thermo Fisher Scientific unless stated otherwise. Fresh CRC tissues from resection were washed with saline water (1% antibiotic-antimycotic), minced into sections (< 1 mm3) and enzymatically digested with collagenase (type II 1 mg/ml and type IV 1 mg/ml) (Sigma-Aldrich) at 37 °C for 30 min. Then the separated cells were centrifuged, resuspended in growth medium (DMEM/F12 (1:1) medium containing 10% fetal bovine serum (FBS), 1X insulin-transferrin-selenium and 1X PS), transferred into a 25-cm2 flask and cultured at 37 °C in a humidified incubator containing 5% CO2. G418 was used for 2 weeks to eliminate fibroblasts. Cell immortalization was performed by hTERT Cell Immortalization Kit (ALSTEM, Richmond, CA, USA). Plate cells in 6-well plate at density of 1 X 105 cells/well. Infect cells with 5μl/well viral supernatant in the presence of 4 μl TransPlus reagent. After 72 h incubation, subculture the cells into 2 X 100 mm dishes and add 2 μg/mL puromycin for stable cell-line generation. Ten days after selection, pick clones for expansion and screen for positive ones. Samples were repeatedly collected for cell species identification and Mycoplasma detection to exclude contamination with other cells or foreign microorganisms.”

**Cell viability measurement**

Cells (2 × 10^3^-3 × 10^3^/well) were plated in 96-well plates. Drug with indicated concentration was added into each well. Following 3 days of drug treatment, cell viability was examined using CellTiter 96 Aqueous One Solution (Promega, Madison, WI, USA). The half inhibitory concentration (IC_50_) values were calculated using GraphPad Prism (version 10.1.1). The initial IC_50_ of the drug, the final IC_50_, and the final drug resistance index were presented in **Supplementary Table 6**.

**Cell proliferation assay**

A concentration of 4000 cells per well were seeded in 96-well plates. Cell proliferation analysis was performed following the instructions of the Incucyte ZOOM Live-Cell Analysis System (Essen BioScience, Ann Arbor, MI, USA).

**Cell Apoptosis Assay**

After indicated treatment, single cell suspension (10^6^) was washed with PBS. Then cells were stained with Binding Buffer, FITC Annexin V and PI from Annexin V-FITC Apoptosis Detection Kit (BD Biosciences). Apoptosis was analyzed on a FACSLyric flow cytometer using FlowJo software. Apoptosis was also analyzed using Caspase-Glo 3/7 Assay (Promega).

**Generation of platinum resistance cell lines**

Cells were treated with oxaliplatin or cisplatin at gradually increased concentrations from IC_10_ to IC_50_. Each concentration was kept for at least 2 weeks till cells reach stable state. Drug resistant cells were maintained in oxaliplatin or cisplatin for a totality of at least 6 months.

**Quantitative real-time PCR**

Total RNA of cell lines and CRC tissues was extracted using RNeasy Mini Kit (Qiagen, Hilden, Germany) or TRIzol reagent (Invitrogen). Reverse transcription and qRT-PCR were perdormed using PrimeScript RT reagent Kit and SYBR® Premix Ex TaqTM GC (Takara, Beijing, China). Primers for qRT-PCR were listed in **Supplementary Table 7**. Relative expression of target genes was calculated versus β-actin and normalized to control groups.

**RNA interference**

SiRNA oligonucleotides were produced by Ribobio. Lipofectamine 3000 Reagent (Invitrogen) was used to deliver siRNAs into cells. ShRNAs for GDIL and CHAC1 were designed and produced by Public Protein/Plasmid Library (Jiangsu, China). ASO targeting GDIL was designed and produced by Ribobio Pharmaceuticals Inc. (Guangzhou, China).

**Lentiviral production and transduction**

HEK293T (5 × 10^5^/well) cells were seeded in 6-cm plate. Plasmids were transduced into HEK293T cells with psPAX2 and pMD2G plasmids. After 72h incubation, supernatant of HEK293T was collected and filtered. Cancer cells were seeded and transduced with lentiviruses and selected with 2 μg/ml puromycin (Thermo Fisher Scientific).

**5’ and 3’ rapid amplification of cDNA ends analysis**

GDIL cDNA ends were performed using the SMARTer RACE 5’/3’ Kit (Clonetech, Palo Alto, CA, USA). Gene-specific primers are presented in **Supplementary Table 7**.

**Evaluation of ROS**

Cells (5 × 10^5^/well) were plated in 6-well plates and harvested in conditioned DMEM containing 20 μM DCFH-DA probe (Millipore Sigma, Burlington, MA, USA) at 37°C for 30 minutes in the dark. Remove DCFH-DA containing medium and wash cells twice with PBS. Immediately subject cells to flow cytometry (FACSLyric, BD Biosciences, San Jose, CA, USA).

**Subcellular fractionation**

Subcellular fractionation of cytoplasm and nuclear of cancer cells was performed using the PARIS Kit (Ambion, Austin, TX, USA). RNA and protein of fractionations were extracted. The primer sequences are listed in **Supplementary Table 7**. The primary antibodies were listed in **Supplementary Table 8**.

**Immunohistochemistry**

Freshly collected tumor samples were fixed with paraformaldehyde (4%) and embedded in paraffin. Paraffin masses were then cut into sections at a thickness of 4 µm. Primary antibodies for staining were listed in **Supplementary Table 8**. Scanning of histological sections were performed with a microscope (Leica Microsystems, Wetzlar, Germany).

**Immunoblotting**

Cells were lysed in a mixture of RIPA lysis buffer (Beyotime) and protease and phosphatase inhibitor cocktail (HY-K0013, MCE, USA). Relative levels of retrieved proteins were analyzed by immunoblot. Primary antibodies and HRP-conjugated secondary antibodies were listed in **Supplementary Table 8**. uncropped original blots were presented in **Supplementary Figure 9-11**.

**Luciferase assays**

CHAC1 3’UTR was cloned into pGL3 vector. Indicated cells (5,000) were seeded into 96-well plates. Cells were transfected with siRNA targeting GDIL or control, luciferase reporters and incubated at 37°C for 48 hours. Cells were lysed and extracts were assayed using Dual luciferase Reporter Assay kit (Promega).
